# Total Chemical Synthesis of RNF4 by Sequential Native Chemical Ligation: C‑To‑N Versus N‑To‑C Strategies

**DOI:** 10.1021/acs.joc.5c03224

**Published:** 2026-02-04

**Authors:** Rajesh Pallava, Saed Bisher, Ashraf Brik

**Affiliations:** Schulich Faculty of Chemistry, Technion – Israel Institute of Technology, 3200008 Haifa, Israel

## Abstract

RNF4, a RING-type E3 ubiquitin ligase, targets polySUMOylated proteins for ubiquitination and subsequent proteasomal degradation. The ability to chemically synthesize RNF4 will enable future studies of its structure and biological function, particularly its role in degrading the oncoprotein PML-RARα in acute promyelocytic leukemia. To achieve this, we performed a total chemical synthesis of RNF4 using sequential native chemical ligation. The presence of nine cysteine residues enables stepwise ligation of five peptide fragments to assemble the full-length protein. Two synthetic strategies were explored: the first employed a convergent C-to-N ligation, while the second used an N-to-C ligation. In the convergent C-to-N approach, cysteine residues were protected with acetamidomethyl groups to prevent side reactions during ligation, although this required multiple deprotection and purification steps. Conversely, the N-to-C synthesis method proceeded efficiently without cysteine protection, thereby simplifying the workflow and reducing the number of purification steps. This research presents a reliable and accessible method for the complete chemical synthesis of RNF4, addressing significant challenges in synthesizing large proteins and opening up new opportunities for future biological research.

## Graphical abstract

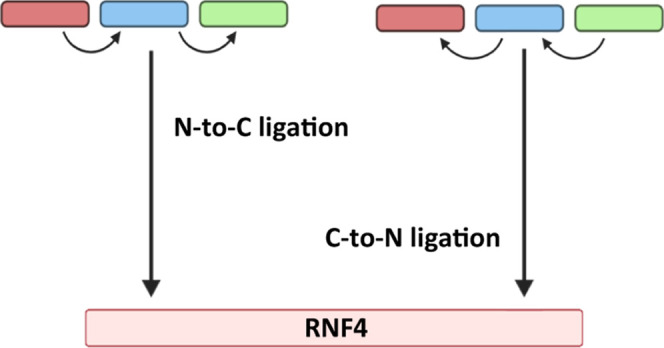

## Introduction

The Ring Finger Protein 4 (RNF4) is an E3 ubiquitin ligase that targets poly-SUMOylated proteins for ubiquitination and subsequent proteasomal degradation.^1^ RNF4 was first identified in the late 1990^s^ and consists of 190 amino acid residues.^2^ The N-terminal region of RNF4 contains a stretch of hydrophobic residues, valine (V), isoleucine (I), and leucine (L), which represent four well-characterized SUMO-interacting motifs (SIMs), designated as SIM1, SIM2, SIM3, and SIM4. These motifs specifically recognize polymeric SUMO chains, a crucial feature required for ubiquitination and subsequent protein degradation.^3^ In addition, RNF4 contains an arginine-rich motif (ARM; residues 73–82), which is essential for recognizing substrates that are simultaneously SUMOylated and phosphorylated.^4^ The C-terminal region of RNF4 harbors a RING domain that mediates ubiquitin transfer from the E2 enzyme to the substrate. This domain adopts a unique cross-brace structure comprising a conserved arrangement of seven cysteine and one histidine residues that coordinate two Zn^2+^ ions, promoting RING dimerization, which is essential for efficient ubiquitin transfer activity.^5^

RNF4 is a SUMO-targeted ubiquitin ligase that links SUMOylation to ubiquitin-dependent proteasomal degradation and plays essential roles in genome stability, DNA repair, and cancer-related signaling.^6^ RNF4 recognizes poly-SUMOylated substrates via SUMO-interacting motifs (SIMs) in its intrinsically disordered N-terminal region.^4^ At the same time, its dimeric C-terminal RING domain activates the E2–ubiquitin conjugate to catalyze direct ubiquitin transfer without forming a covalent E3-ubiquitin intermediate.^7^ In acute promyelocytic leukemia (APL), arsenic trioxide (As_2_O_3_) induces poly-SUMOylation of the PML protein, leading to RNF4 recruitment and proteasomal degradation of PML.^8^ In addition to its role in protein degradation, RNF4 has also been implicated in the stabilization and transcriptional activation of several oncogenic proteins essential for cell survival, including c-Myc, c-Jun, β-catenin, and the Notch intracellular domain (N-ICD).^9^

Previous studies have shown that RNF4 activity is regulated by two post-translational modifications (PTMs): methylation and phosphorylation. Methylation of RNF4 at Arg164 by protein arginine methyltransferase 5 inhibits its interaction with PML-RARα, thereby stabilizing PML-RARα and promoting drug resistance.^10^ Conversely, phosphorylation of RNF4 by cyclin-dependent kinases (CDKs) at Thr26 and Thr112 enhances its activity toward MDC1, which is required for its degradation and proper homologous recombination repair during S phase.^11^ However, it remains unclear whether these PTMs affect RNF4 function within PML nuclear bodies (PML-NBs). The chemical synthesis of RNF4 could provide a powerful opportunity to investigate how these modifications regulate RNF4 activity in PML-NBs. Therefore, there is a clear need for a robust and reliable route to the total chemical synthesis of RNF4. To the best of our knowledge, no reports to date describe the total chemical synthesis or semisynthesis of RNF4. Until now, RNF4 has been obtained only through recombinant expression,^2^ which is itself difficult, particularly due to handling issues and the need to introduce mutations to enable expression and support biological studies.^10,11^

In this study, we report our initial efforts toward the chemical synthesis of the human RNF4 protein, which comprises 190 amino acid residues, including nine cysteines. We examine two synthetic strategies investigated: a C-to-N approach and an N-to-C approach (Figure 1). Based on the protein sequence, RNF4 was divided into five peptide fragments for each strategy, which were subsequently ligated by native chemical ligation (NCL) to get the full-length RNF4 protein. Initially, we pursued the C-to-N ligation strategy, employing Acetamidomethyl (Acm) protection of cysteine (Cys) residues in two peptide fragments to suppress side reactions during ligation. However, this approach required multiple Acm deprotection and purification steps, resulting in substantial material loss and a reduced overall yield of the RNF4 protein. In contrast, the N-to-C strategy employed cysteine-unprotected fragments and proceeded more efficiently, requiring fewer purification steps and ultimately providing an improved overall yield.

**Figure 1:**
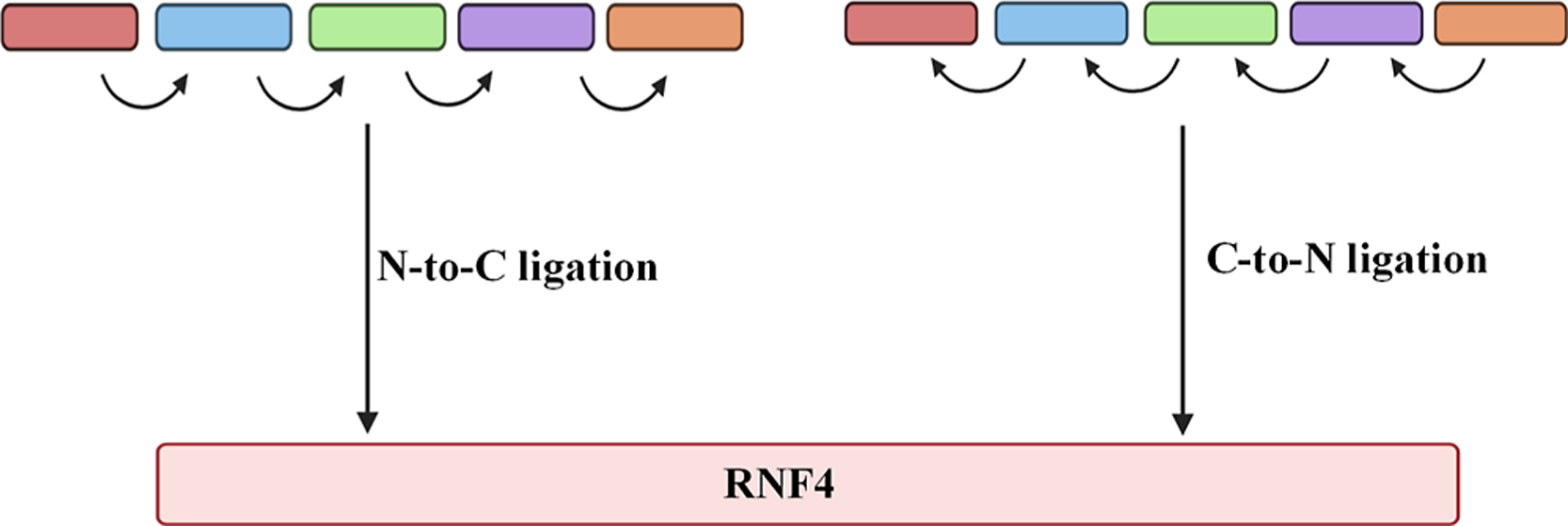
Schematic representation of N-to-C and C-to-N ligation strategies.

## Results and Discussion

Synthetic strategies for preparing complex proteins are essential for generating novel proteins for diverse applications.^12^ Solid-phase peptide synthesis (SPPS)^13^ and chemoselective ligation technologies have revolutionized protein synthesis by enabling efficient assembly of proteins for structural and functional studies.^14^ Native chemical ligation (NCL) is widely used to join unprotected peptide segments via a C-terminal thioester and an N-terminal cysteine.^15^ To address the growing demand for challenging multistep protein syntheses, several advanced strategies have been developed to improve homogeneity and scalability.^16^ Furthermore, the ligation methods, such as solid-supported ligation, convergent ligation, and one-pot approaches, and the fragment sequence strongly affect synthesis time, yield, and product purity.^17–22^ These methods enable the efficient synthesis of proteins with increasing size and complexity. To chemically prepare RNF4, we initially explored the C-to-N approach based on sequence considerations and the synthetic accessibility of the peptide fragments. This strategy allowed us to systematically evaluate the feasibility of sequential ligation starting from the C-terminal segments before adopting a convergent assembly strategy. The full-length sequence was divided into five peptide fragments: Fragment **1a** Cys-RNF4(160–190); Fragment **2a** Cys­(Acm)-RNF4(133–158)-NHNH_2_; Fragment **3a** Cys­(Acm)-RNF4(92–131)-MMP; Fragment **4a** Cys-RNF4(52–90)-NHNH_2_; and fragment **5a** Nle-RNF4(2–50)-MMP (Scheme 1). Fragments **2a** and **3a** were synthesized with an N-terminal Cys­(Acm) protecting group to prevent undesired intramolecular cyclization during NCL. Fragments **3a** and **5a** were prepared as thioesters using the *N*-acyl-benzimidazolinone (Nbz) strategy.^23^ All peptide fragments were synthesized by Fmoc-SPPS, purified, and obtained in good yields (Supporting Information Figures S1–S7).

**Scheme 1:**
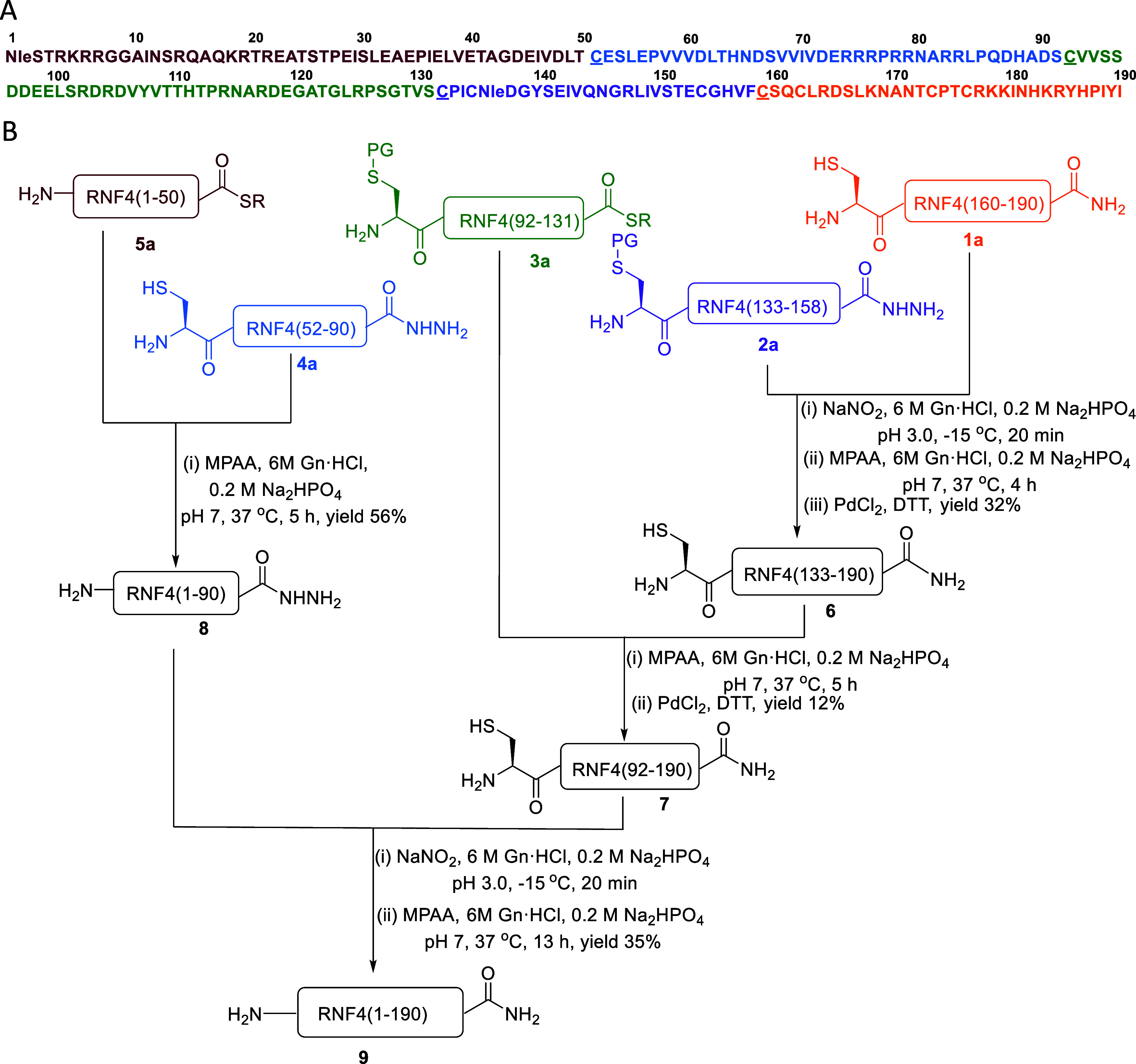
Total Chemical Synthesis of RNF4; (A) Primary Sequence of RNF4 with Ligation Sites Highlighted in Red; The Met-1 and Met-136 Residues Were Substituted With the Oxidation-Resistant Norleucine (Nle) Analogues; (B) Synthetic Route of RNF4 Using the C-To-N (Convergent) Ligation Strategy; PG- Acm­(Acetamidomethyl), R = MMP­(Methyl 3-Mercaptopropionate)

After preparing all peptide fragments, the synthesis was initiated by converting fragment **2a**, Cys­(Acm)-RNF4(133–158)-NHNH_2_, to the corresponding acyl azide using NaNO_2_ ^21b^ in 6 M Gn·HCl, 0.2 M Na_2_HPO_4_ buffer at pH 3.0 and −15 °C for 20 min. A solution of MPAA was prepared in a similar buffer at pH 7 and added to the reaction mixture to generate the thioester intermediate in situ. This activated species was then ligated with fragment **1a**, Cys-RNF4­(160–190), and the reaction mixture was incubated at 37 °C for 40 min. The reducing agent TCEP was then added, and the reaction was allowed to proceed for an additional 2 h (Scheme 1). The progress of the ligation was monitored by analytical HPLC, which confirmed the formation of the desired peptide **6** after 4 h. Subsequently, the peptide underwent smooth Acm deprotection with PdCl_2_ at 37 °C for 1 h, quenched with DTT, and was purified.^24^ to give the ligated peptide **6** in ∼32% yield (Supporting Information Figure S8). Before the subsequent ligation, peptide **6** was dissolved in 6 M Gn·HCl, 0.2 M Na_2_HPO_4_, pH 7, containing MPAA and TCEP, and this solution was added to fragment **3a** Cys (Acm)-RNF4(92–131)-MMP.

The mixture was incubated at 37 °C for 3 h, during which analytical HPLC confirmed formation of the desired ligated peptide **7**. The N-terminal Acm protecting group was then deprotected with PdCl_2_ for 1 h, followed by quenching with DTT, washing, and HPLC purification to give ligated product **7** in ∼12% yield (Supporting Information Figure S9). The ligation of fragment **5a** Nle-RNF4(2–50)-MMP with fragment **4a** Cys-RNF4(52–90)-NHNH_2_ was performed using a similar strategy. Fragment **4a** was dissolved in 6 M Gn·HCl, 0.2 M Na_2_HPO_4_, pH 7, containing TCEP and MPAA, and the resulting solution was added to fragment **5a**. The mixture was incubated at 37 °C for 5 h and monitored by analytical HPLC. After completion, the crude mixture was purified, and peptide **8** was isolated (Supporting Information Figure S10) in 56% yield. We employed the same strategy for the final ligation between peptides **8** and **7** (Figure 2A). Peptide **8**, RNF4(1–90)-NHNH_2_, was first converted to the corresponding acyl azide using NaNO_2_ in 6 M Gn·HCl, 0.2 M Na_2_HPO_4_ buffer, pH 3.0, at −15 °C for 20 min. A solution of MPAA was prepared in the same buffer, adjusted to pH 7, and added to the reaction mixture to generate the thioester intermediate in situ. This activated thioester was then ligated with peptide **7**, Cys-RNF4(92–190). The reaction mixture was incubated at 37 °C for 40 min. TCEP was then added, and the reaction was allowed to proceed for an additional 4 h (Supporting Information Figure S11). Analytical HPLC confirmed formation of the full-length RNF4 product **9** after 5 h. The ligated polypeptide was isolated in ∼35% yield. Consequently, we performed Western blotting with an anti-RNF4 antibody to further verify the integrity of the final product and to demonstrate that our chemically synthesized RNF4 is correctly recognized by the anti-RNF4 antibody (Figure 2B,C).

**Figure 2:**
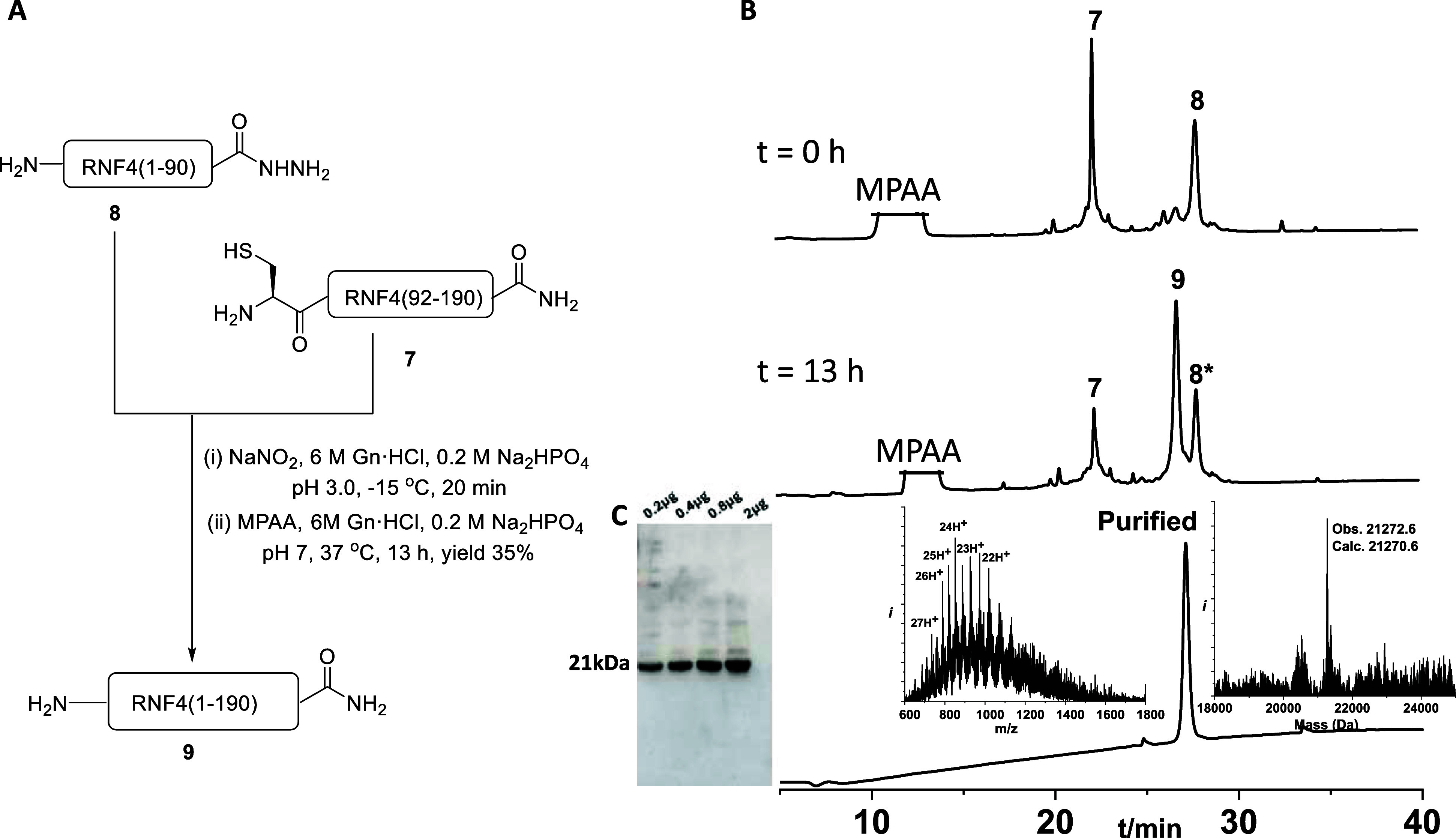
(A) General schematic of the final C-to-N (convergent) ligation between peptides **8** and **7**; (B) analytical HPLC and ESI-MS of the final NCL product RNF4 (observed mass: 21,272.6 ± 2 Da; expected mass: 21,270.6 Da); (C) Western blot analysis using anti-RNF4 antibody, 8* = hydrolysis.

After completing the RNF4 synthesis using the C-to-N ligation strategy, we found that repeated purification and Acm deprotection steps made the process time-consuming and reduced the overall yield. To address these limitations, we explored an alternative N-to-C ligation strategy to minimize purification steps, streamline the assembly, and improve the overall efficiency and yield.

We pursued the synthesis of RNF4 using an N-to-C ligation strategy based on hydrazide chemistry (Scheme 2). This approach utilizes cysteine-free peptide hydrazides that undergo chemoselective ligation with Cys-containing peptides to form native amide bonds. Importantly, peptide hydrazides are readily accessible via standard Fmoc-based solid-phase peptide synthesis (SPPS). For this purpose, we initially prepared three fragments using CTC-hydrazine resin.^25^

**Scheme 2:**
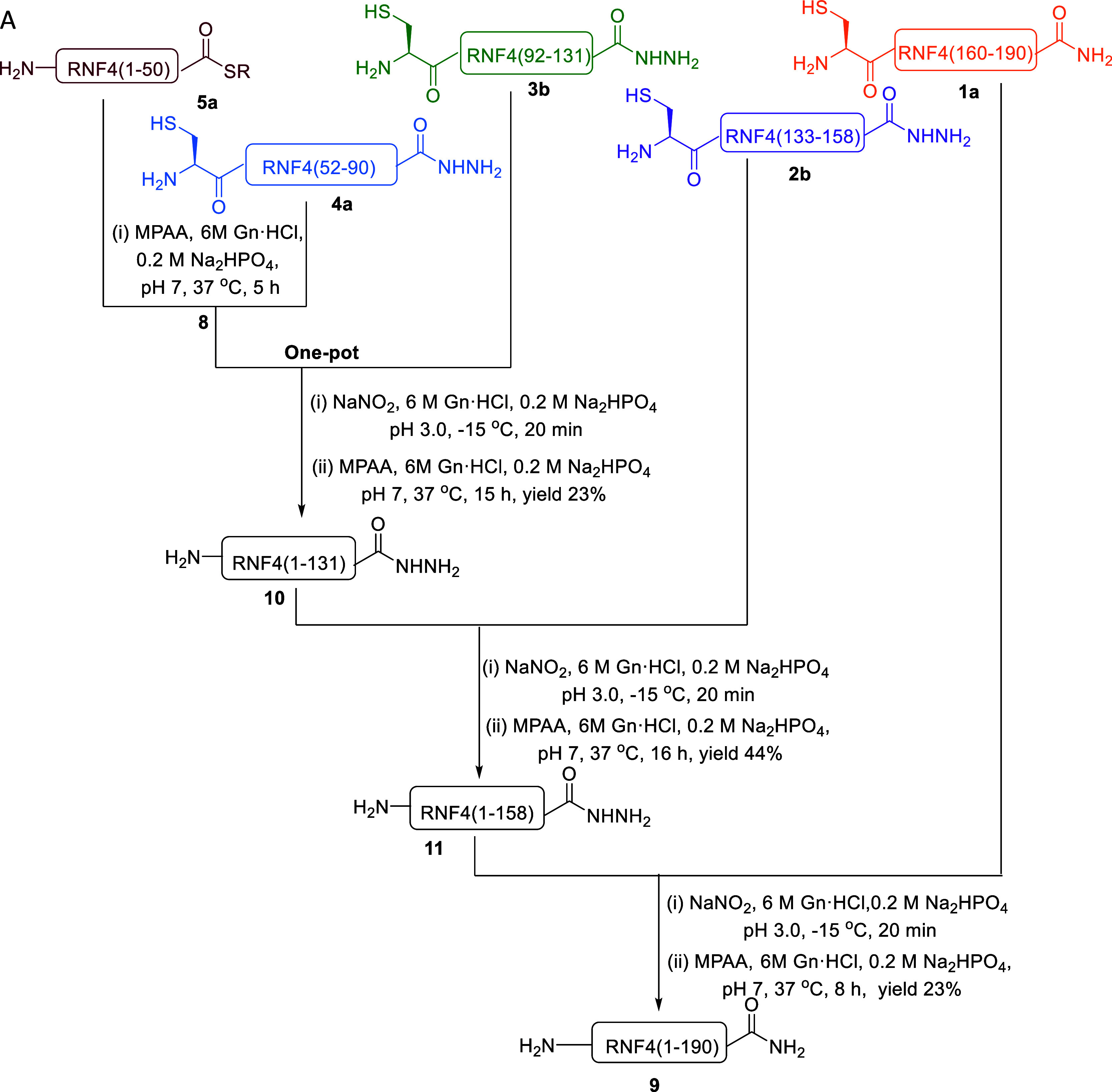
N-To-C Ligation Approach for the Total Chemical Synthesis of RNF4; (A) Synthetic Route of RNF4 Using the N-To-C Ligation Strategy, R = MMP (Methyl 3-Mercaptopropionate)

However, during the synthesis of fragment **3b** using the CTC-hydrazine resin, we observed severe peptide aggregation that hampered further chain elongation. In light of our report and Hartrampf’s recent work^26^ on the effect of the MeDbz linker on reducing aggregation during SPPS of complex peptides, we switched from CTC-hydrazine resin to a Dbz linker coupled to Rink amide resin. Using this strategy, fragment **3b** (Cys-RNF4(92–131)) was successfully synthesized by SPPS with excellent purity. Subsequent Dbz-to-Nbz cyclization was performed, followed by conversion of the resulting Nbz moiety to the corresponding hydrazide using 10% hydrazine.^27^ Finally, Acm deprotection with PdCl_2_ afforded the desired fragment **3b** (Figure 3C)

**Figure 3:**
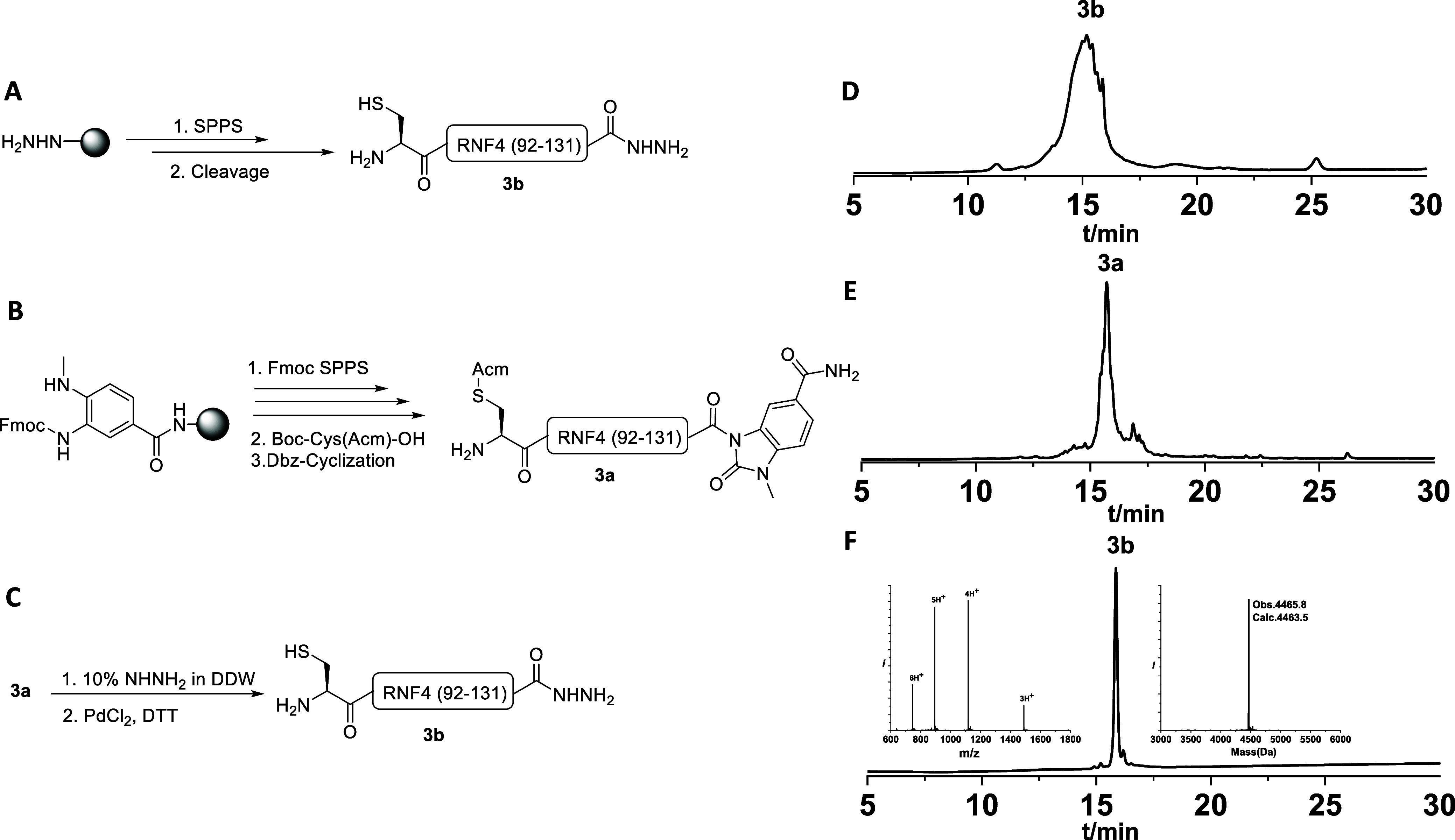
Synthesis of the same peptide sequence using (A) solid-phase peptide synthesis using a CTC-hydrazine resin. (B) solid-phase peptide synthesis using a Dbz linker. (C) Conversion of peptide **3a** to **3b** via hydrazinolysis (NHNH_2_). (D) Crude analytical HPLC of the peptide synthesized on CTC-hydrazine resin. (E) Crude analytical HPLC of the peptide synthesized using the Dbz linker. (F) Analytical HPLC of purified peptide **3b**.

With all fragments in hand, we initiated the synthesis of RNF4 using the N-to-C ligation strategy (Scheme 2). We first performed a one-pot ligation of three fragments: **5a**, **4a**, and **3b**. Fragment **4a** was dissolved in 6 M Gn·HCl, 0.2 M Na_2_HPO_4_, pH 7, containing TCEP and MPAA, and the resulting solution was added to fragment **5a**. The reaction mixture was incubated at 37 °C and monitored by analytical HPLC, which confirmed the formation of the desired ligated peptide **8** after 5 h (Supporting Information Figure S12). Before the subsequent ligation, the reaction mixture was desalted, and the pH was adjusted to ∼3 using 2 N HCl. The intermediate **8** was then converted to the corresponding peptide thioester by treatment with NaNO_2_ in 6 M Gn·HCl, 0.2 M Na_2_HPO_4_ buffer at pH 3.0 at −15 °C for 20 min.

Following this activation, MPAA was added to 6 M Gn·HCl, 0.2 M Na_2_HPO_4_ buffer at pH 7 to generate the thioester intermediate in situ. The resulting activated thioester was then ligated with fragment **3b**, Cys-RNF4(92–131)-NHNH_2_, and the reaction mixture was incubated at 37 °C for 40 min. TCEP was then added, and the ligation was allowed to proceed for an additional 15 h. Reaction progress was monitored by analytical HPLC, which confirmed formation of the desired ligated peptide **10**, along with the hydrolysis byproduct. The crude reaction mixture was purified by preparative HPLC, affording the isolated peptide in 23% yield (Supporting Information Figure S12). The next ligation between peptide **10**, RNF4(1–131)-NHNH_2_, and fragment **2b** was carried out by first converting **10** to the corresponding acyl azide using NaNO_2_ in 6 M Gn·HCl, 0.2 M Na_2_HPO_4_ buffer at pH 3 at −15 °C for 20 min, followed by the addition of MPAA as described before. The resulting peptide thioester was then reacted with fragment **2b**, and the reaction mixture was incubated at 37 °C for an additional 15 h. Analytical HPLC confirmed formation of the desired ligated peptide **11** (Scheme 2). Preparative HPLC was used to purify the crude reaction mixture, affording the isolated peptide in 44% yield (Supporting Information Figure S13). For the final ligation, we employed the same strategy, initiating the reaction between peptide **11** and **1a**. Peptide **11** was first converted to the corresponding acyl azide using NaNO_2_ in 6 M Gn·HCl, 0.2 M Na_2_HPO_4_ buffer at pH 3.0 at −15 °C for 20 min, followed by the addition of MPAA as described before. The resulting peptide thioester was then reacted with fragment **1a**, and the reaction mixture was incubated at 37 °C for an additional 8 h (Figure 4A). Reaction progress was monitored by analytical HPLC, which confirmed formation of the desired full-length RNF4 product **9**. The crude reaction mixture was finally purified by preparative HPLC, affording the isolated product in 23% yield (Figure 4B).

**Figure 4:**
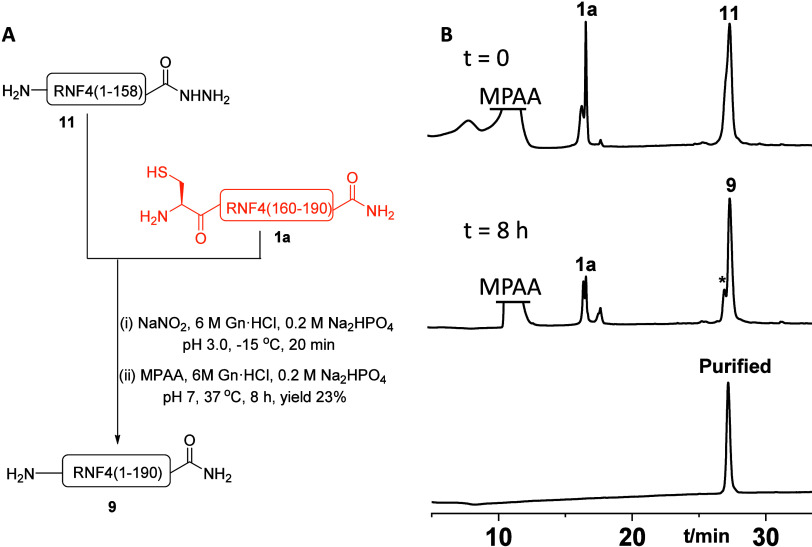
(A) General schematic of the final N-to-C ligation between peptides **11** and **1a**; (B) analytical HPLC and ESI-MS of the final NCL product RNF4. *Represent unidentified mass.

Although the full-length protein was assembled and recognized by the Anti-RNF4 antibody, we first attempted to solubilize it in aqueous buffers to investigate its secondary structure. RNF4 was dissolved in 8 M Gn·HCl and then dialyzed against a degassed buffer containing 20 mM Tris, 1 mM NaCl, 1 mM DTT, and 50 μM ZnCl_2_. Multiple alternative buffer solvents, such as sodium phosphate and HEPES, were also explored in the presence of Zn^2+^ at varying μM concentrations, various redox reagents, and glycerol, but none improved refolding. We further explored a segmental folding strategy using peptide **7**, but this approach likewise failed to yield successful folding. However, in all cases, RNF4 precipitated, formed highly aggregated species, failed to fold correctly, and exhibited a low Circular Dichroism signal, indicating limited solubility and intrinsic instability in aqueous solution. We attributed this to the structurally disordered region in RNF4, prompting us to explore alternative strategies to stabilize it, which we are currently pursuing.

Despite this, both synthetic approaches, the C-to-N and the N-to-C, were successful; however, they differed in efficiency. The C-to-N strategy was considerably more time-consuming for synthesizing RNF4, primarily because of the requirement for Acm cysteine deprotection and the associated multiple purification steps after intermediate transformations. These additional steps not only prolonged the overall synthesis but also led to cumulative material losses, reducing the overall yield. Using the C-to-N approach, full-length RNF4 could ultimately be isolated, yielding ∼1.3% (1.2 mg) based on the yield of each ligation. In contrast, the N-to-C approach proved more efficient because it did not require Cys protection, thereby eliminating the need for deprotection steps. This strategy enabled a more streamlined workflow, with fewer reaction steps and significantly fewer purification cycles, affording the isolated product with an improved overall yield of ∼2.3% (0.45 mg).

## Conclusions

We accomplished the total chemical synthesis of RNF4 from five peptide fragments using both C-to-N (convergent) and N-to-C ligation strategies. Based on two independent syntheses, our results demonstrate that the N-to-C approach offers advantages for complex protein targets, with reduced reaction time, fewer purification steps, and fewer than the C-to-N (convergent) strategy. To the best of our knowledge, this is the first study to systematically evaluate synthetic routes for preparing the RNF4 protein. These findings provide valuable guidelines for the synthesis of modified RNF4 analogues, such as methylated and phosphorylated variants, and offer a versatile, scalable strategy for generating RNF4 derivatives for biochemical and biomedical investigations.

## Experimentalsection

### General Reagents

Fmoc-SPPS was performed either manually in Teflon-filtered syringes purchased from Torviq or using an automated peptide synthesizer (CS336X, CSBIO). 2-Chlorotrityl chloride (2-CTC) resin was purchased from Chem-Impex. All protected amino acids were purchased from Chem-Impex and Iris-Biotech. The activating reagents [(6-*c* 6-chlorobenzotriazolyl)­oxy­(dimethylamino)­methylidene]­dimethylazaniumhexafluorophosphate (HCTU), 1-[bis­(dimethylamino)­methylene]-1*H*-1,2,3-triazolo [4,5-*b*] pyridinium 3-oxid hexafluorophosphate (HATU), and 1- 1-hydroxybenzotriazole monohydrate (HOBt) were purchased from Luxembourg Bio Technologies. All solvents, including *N*,*N*-dimethylformamide (DMF), dichloromethane (DCM), acetonitrile (ACN), *N*,*N*-diisopropylethylamine (DIEA), Piperidine, Diethyl ether (Et_2_O), and Trifluoroacetic acid (TFA), were purchased from Bio-Lab. Triisopropyl silane (TIPS) was purchased from Sigma-Aldrich. Paraformaldehyde, 4% in PBS, was purchased from Affymetrix. 4–20% MOPS gel (MP42G12) was purchased from Merck. Additional miscellaneous chemicals were purchased from Merck, Strem Chemicals, and Alfa Aesar.

#### Caution

TFA, DMF, DCM, ACN, Et_2_O, and coupling reagents (HCTU, HATU, and HOBt) are hazardous chemicals with high toxicity and potential health risks. All procedures involving these reagents were performed in a well-ventilated fume hood, with appropriate personal protective equipment (PPE) including lab coats, chemical-resistant gloves, and safety goggles.

### List of the Protected Amino Acids Used in Peptide Synthesis

Fmoc-Ala-OH, Fmoc-Asp­(OtBu)–OH, Fmoc-Gly-OH, Fmoc-Glu­(OtBu)–OH, Fmoc-His­(Trt)–OH, FmocIle-OH, Fmoc-Leu-OH, Fmoc-Phe-OH, Fmoc-Asn­(Trt)–OH, Fmoc-Gln­(Trt)–OH, Fmoc-Arg­(Pbf)–OH, Fmoc- Lys­(Boc)–OH, Fmoc-Pro-OH, Fmoc-Tyr­(*t*Bu)–OH, Fmoc-Ser­(*t*Bu)–OH, Fmoc-Thr­(*t*Bu)–OH, Fmoc-Asp­(OtBu)- OH, Fmoc-Cys­(Trt)–OH, Fmoc-Nle–OH, Fmoc-Val-OH, Fmoc-Asp­(OtBu)­Thr­(ψMe, MePro)–OH, Fmoc-(Dmb)­Gly-OH, Fmoc-Leu-Ser­(ψMe, MePro)–OH.

### Peptide Synthesis, Purification, and Analysis

RNF4 fragments were synthesized on Rink amide MBHA and 2-chlorotrityl chloride (CTC) resins using a synthesizer (CSBio CS336X automated peptide synthesizer). After swelling in DMF for 1 h, Fmoc deprotection was achieved by adding 20% piperidine in DMF for 2 × 5 min cycles. The resin was washed three times with DMF, and amino acid^17^ (AA) was added for 45 min (4 eq AA, 4 eq HCTU, 8 eq DIPEA). When twin couplings occurred, the coupling period decreased to 2 × 30 min. The coupling phase for dipeptides involved 2.5 eq. AA, 2.5 eq. HATU, and 5 eq. DIPEA. Underlined AAs were coupled as dipeptides, while bold AAs were coupled twice. The N-terminal amino acid in the sequence was used in its Boc- and Acm-protected forms. The peptide was cleaved from the resin after 3 h in a cleavage solution (95% TFA, 2.5% H_2_O, 2.5% TIPS). The peptide was then precipitated with cold diethyl ether. After centrifugation (4000 rpm, 15 min, 4 °C), the pellet was dissolved in 50% ACN in H_2_O and lyophilized.

The lyophilized peptide was dissolved in 50% ACN in H_2_O and purified by preparative high-performance liquid chromatography (HPLC) on a Dionex Ultimate 3000 system (Thermo Scientific). H_2_O + 0.05% TFA and ACN +0.05% TFA were used as buffers A and B, respectively. Gradients from 0 to 65% B over 10–45 min were used at a flow rate of 15 mL/min. The peptide mass was confirmed by LCQ Fleet Ion Trap (Thermo Scientific), and purity was assessed by analytical HPLC (0–60% B in 30 min, 1.2 mL/min), using the same solvents as for preparative HPLC.

**For resin N**-**MeNbz formation^23^**, the peptide-N-MeDbz-resin was treated with 4-nitrophenyl chloroformate (5 equiv) in DCM while shaking for 1 h at room temperature, repeated 3 times. The resin was drained and treated with a 0.5 M DIEA solution in DMF for 30 min to complete Nbz formation, repeated 3 times. Finally, the resin was washed with DCM and dried under vacuum.

## Supplementary Material



## Data Availability

The data underlying this study are available in the published article and its Supporting Information.

## Notes

Synthesis and characterization of all compounds, solid-phase chemical synthesis of peptide fragments, and native chemical ligation of peptides (PDF)

## References

[1] Sriramachandran A. M., Dohmen R. J. (2014). SUMO-targeted ubiquitin ligases. Biochim. Biophys. Acta Gen. Subj..

[2] Chiariotti L., Benvenuto G., Fedele M., Santoro M., Simeone A., Fusco A., Bruni C. B. (1998). Identification and
Characterization of a Novel RING-Finger Gene (RNF4) Mapping at 4p16.3. Genomics.

[3] Tatham M. H., Geoffroy M. C., Shen L., Plechanovova A., Hat-tersley N., Jaffray E. G., Palvimo J. J., Hay R. T. (2008). RNF4 is
a poly-SUMO-specific E3 ubiquitin ligase required for arsenic-induced
PML degradation. Nat. Cell Biol..

[4] Kuo C. Y., Li X., Kong X. Q., Luo C., Chang C. C., Chung Y., Shih H. M., Li K. K., Ann D. K. (2014). An Arginine-Rich
Motif of Ring Finger Protein 4 (RNF4)
Oversees the Recruitment and Degradation of the Phosphorylated and
SUMOylated Krüppel-Associated Box Domain-Associated Protein
1 (KAP1)/TRIM28 Protein during Genotoxic Stress. J. Biol. Chem..

[5] Plechanovova A., Jaffray E. G., Tatham M. H., Naismith J. H., Hay R. T. (2012). Structure
of a RING E3 ligase and ubiquitin-loaded E2 primed for catalysis. Nature.

[6] Her J., Zheng H., Bunting S. F. (2024). RNF4 sustains
Myc-driven tumorigenesis
by facilitating DNA replication. J. Clin. Invest..

[7] Pickart C. M., Eddins M. J. (2004). Ubiquitin: structures,
functions,
mechanisms. Biochim. Biophys. Acta Gen. Subj..

[8] Lallemand-Breitenbach V., Jeanne M., Benhenda S., Nasr R., Lei M., Peres L., Zhou J., Zhu J., Raught B., de Thé H. (2008). Arsenic degrades
PML or PML-RARα
through a SUMO-triggered RNF4/ubiquitin-mediated pathway. Nat. Cell Biol..

[9] Thomas J. J., Abed M., Heuberger J., Novak R., Zohar Y., Beltran Lopez A. P., Trausch-Azar J. S., Ilagan M. X. G., Benhamou D., Dittmar G., Kopan R., Birchmeier W., Schwartz A. L., Orian A. (2016). RNF4-Dependent
Oncogene Activation by Protein Stabilization. Cell Rep..

[10] Huang X., Yang Y., Zhu D., Zhao Y., Wei M., Li K., Zhu H. h., Zheng X. (2022). PRMT5-mediated RNF4 methylation promotes
therapeutic resistance of APL cells to As_2_O_3_ by stabilizing oncoprotein PML-RARα. Cell. Mol. Life Sci..

[11] Luo K., Deng M., Li Y., Wu C., Xu Z., Yuan J., Lou Z. (2015). CDK-mediated RNF4 phosphorylation
regulates homologous recombination in S-phase. Nucleic Acids Res..

[12] Bondalapati S., Jbara M., Brik A. (2016). Expanding
the chemical toolbox for the synthesis of large and uniquely modified
proteins. Nat. Chem..

[13] Merrifield R. B. (1963). Solid Phase
Peptide Synthesis. I. The Synthesis of a Tetrapeptide. J. Am. Chem. Soc..

[14] Dawson P. E., Kent S. B. (2000). Synthesis of native
proteins by chemical ligation. Annu. Rev. Biochem..

[15] Dawson P. E., Muir T. W., Clarklewis I., Kent S. B. H. (1994). Synthesis of
Proteins by Native Chemical Ligation. Science.

[16] Conibear A.
C., Watson E. E., Payne R. J., Becker C. F. W. (2018). Native chemical ligation in protein
synthesis and semi-synthesis. Chem. Soc. Rev..

[17] Bang D., Kent S. B. (2004). A one-pot total synthesis of crambin. Angew. Chem., Int. Ed..

[18] Jbara M., Maity S. K., Morgan M., Wolberger C., Brik A. (2016). Chemical Synthesis of Phosphorylated
Histone H2A at Tyr57 Reveals Insight into the Inhibition Mode of the
SAGA Deubiquitinating Module. Angew. Chem.,
Int. Ed..

[19] Li J., Li Y., He Q., Li Y., Li H., Liu L. (2014). One-pot native
chemical ligation of peptide hydrazides enables total synthesis of
modified histones. Org. Biomol. Chem..

[20] Fauvet B., Butterfield S. M., Fuks J., Brik A., Lashuel H. A. (2013). One-pot
total chemical synthesis of human alpha-synuclein. Chem. Commun..

[21] Zuo C., Zhang B., Yan B., Zheng J. S. (2019). One-pot
multi-segment condensation strategies for chemical
protein synthesis. Org. Biomol. Chem..

[22] Liu S., Pentelute B. L., Kent S. B. (2012). Convergent chemical synthesis of [lysine­(24,38,83)]
human erythropoietin. Angew. Chem. Int. Ed..

[23] Blanco-Canosa J. B., Nardone B., Albericio F., Dawson P. E. (2015). Chemical Protein
Synthesis Using a Second-Generation N-Acylurea Linker for the Preparation
of Peptide-Thioester Precursors. J. Am. Chem.
Soc..

[24] Maity S. K., Jbara M., Laps S., Brik A. (2016). Efficient Palladium-Assisted
One-Pot Deprotection of (Acetamido-methyl) Cysteine Following Native
Chemical Ligation and/or Desulfurization To Expedite Chemical Protein
Synthesis. Angew. Chem., Int. Ed..

[25] Zheng J. S., Tang S., Guo Y., Chang H. N., Liu L. (2012). Synthesis
of Cyclic Peptides and Cyclic Proteins via Ligation of Peptide Hydrazides. ChemBioChem.

[26] Bondalapati S., Eid E., Mali S. M., Wolberger C., Brik A. (2017). Total chemical synthesis
of SUMO-2-Lys63-linked
diubiquitin hybrid chains assisted by removable solubilizing tags. Chem. Sci..

[27] Selvaraj A., Chen H. T., Ya-Ting
Huang A., Kao C. L. (2018). Expedient on-resin
modification of a peptide C-terminus through a benzotriazole linker. Chem. Sci..

