# Supplementary material for: Total Chemical Synthesis of RNF4 by Sequential Native Chemical Ligation: C‑To‑N Versus N‑To‑C Strategies
Source: J Org Chem. 2026 Feb 4;91(7):2965–72. doi: 10.1021/acs.joc.5c03224 (PMC12930501; doi:10.1021/acs.joc.5c03224)
Supplement: Supplementary file 1 [file jo5c03224_si_001.pdf]

Supporting Information

Total Chemical Synthesis of RNF4 by Sequential Native Chemical Ligation: C-to-N versus N-to-C Strategies

Rajesh Pallava[#a] , *Saed Bisher*[#a], and Ashraf *Brik*[*a]

[a] Schulich Faculty of Chemistry, Technion – Israel Institute of Technology, Haifa, Israel

# contributed equally

Correspondence to: abrik@technion.ac.il

Table of Contents

1. General reagents S3
2. List of the protected amino acids used in peptide synthesis S3
3. Peptide synthesis, purification, and analysis S4
4. RNF4 sequences S4
5. Synthesis of Fragment-(1a) Cys-RNF4(160–190) S5
6. Synthesis of Fragment-(2a) Cys (Acm)-RNF4(133-158)-$NHNH_2$ S6
7. Synthesis of Fragment-(2b) Cys-RNF4(133-158)-$NHNH_2$ S7
8. Synthesis of Fragment-(3a) Cys (Acm)-RNF4(92-131)-MMP S8
9. Synthesis of Fragment-(3b) Cys-RNF4(92-131)- $NHNH_2$ S10
10. Synthesis of Fragment-(4a) Cys-RNF4(52-90)-$NHNH_2$ S12
11. Synthesis of Fragment-(5a) Nle-RNF4(1-50)-MMP S14
12. General procedure for Native chemical ligation (NCL) S14
13. NCL of fragment-1a and fragment-2a S15
14. NCL of peptide-6 and fragment-3a S17
15. NCL of fragment 4a and fragment 5a S19
16. NCL of peptide 7 and peptide 8 S21
17. One-pot ligation of fragments 5a-3b S23
18. NCL of peptide 10 and fragment 2b S25
19. NCL of peptide 11 and fragment 1a S27

1. General reagents

Solid-phase peptide synthesis (SPPS) was performed either manually using Teflon-filtered syringes purchased from Torviq or with an automated peptide synthesizer (CS336X, CSBIO). 2-Chlorotrityl chloride (2-CTC) resin was obtained from Chem-Impex. All protected amino acids were purchased from Chem-Impex and Iris-Biotech. The activating reagents, [(6-chlorobenzotriazol1yl)oxy(dimethylamino)methylidene]dimethylazaniumhexafluorophosphate (HCTU), 1-[Bis(dimethylamino)methylene]-1H-1,2,3-triazolo [4,5-b] pyridinium 3-oxid hexafluorophosphate (HATU), and 1-hydroxybenzotriazole monohydrate (HOBt), were purchased from Luxembourg Bio Technologies. All solvents, including N,N-dimethylformamide (DMF), dichloromethane (DCM), acetonitrile (ACN), N,N-diisopropylethylamine (DIEA), piperidine, diethyl ether (Et_2O), and trifluoroacetic acid (TFA), were purchased from Bio-Lab. Triisopropylsilane (TIPS) was obtained from Sigma-Aldrich. Paraformaldehyde, 4% in PBS, was obtained from Affymetrix. 4-20% MOPS gel (MP42G12) was purchased from Merck. RNF4 Antibody (205F2G) purchased from Santa Cruz. Additional miscellaneous chemicals were acquired from Merck, Strem Chemicals, and Alfa Aesar.

Caution: TFA, DMF, DCM, ACN, Et_2O, and coupling reagents (HCTU, HATU, and HOBt) are hazardous chemicals with high toxicity and health risks. All procedures involving these reagents should be performed in a well-ventilated fume hood, using appropriate personal protective equipment (PPE) such as lab coats, chemical-resistant gloves, and safety goggles.

2. List of the protected amino acids used in peptide synthesis

Fmoc-Ala-OH, Fmoc-Asp(OtBu)-OH, Fmoc-Gly-OH, Fmoc-Glu(OtBu)-OH, Fmoc-His(Trt)-OH, Fmoc-Ile-OH, Fmoc-Leu-OH, Fmoc-Phe-OH, Fmoc-Asn(Trt)-OH, Fmoc-Gln(Trt)-OH, Fmoc-Arg(Pbf)-OH, Fmoc-Lys(Boc)-OH, Fmoc-Pro-OH, Fmoc-Tyr(tBu)-OH, Fmoc-Ser(tBu)-OH, Fmoc-Thr(tBu)-OH, Fmoc-Asp(OtBu)-OH, Fmoc-Cys(Trt)-OH, Fmoc-Nle-OH, Fmoc-Val-OH, Fmoc-Asp(OtBu)-Thr(ψMe,MePro)-OH, Fmoc-(Dmb)Gly-OH, and Fmoc-Leu-Ser(ψMe,MePro)-OH.

3. Peptide Synthesis, Purification, and Analysis

RNF4 fragments were synthesized using Rink amide MBHA and 2-chlorotrityl chloride (CTC) resin on a CSBio CS336X automated peptide synthesizer. After swelling in DMF for 1 hour, Fmoc deprotection was performed by adding 20% piperidine to DMF for two 5-minute cycles. The resin was washed three times with DMF, and amino acid (AA) was added for 45 minutes (4 eq AA, 4 eq HCTU, 8 eq DIPEA). When double couplings were needed, the coupling time was reduced to two 30-minute cycles. The coupling phase for dipeptides used 2.5 eq AA, 2.5 eq HATU, and 5 eq DIPEA. Underlined AAs were coupled as dipeptides, while bold AAs were coupled twice. The final amino acid in the sequence was used in its Boc- and Acm-protected form. The peptide was cleaved from the resin after 3 hours in a cleavage solution of 95% TFA, 2.5% H_2O, and 2.5% TIPS. Afterwards, the peptide was precipitated with cold diethyl ether. Following centrifugation at 4000 rpm for 15 minutes at 4°C, the pellet was dissolved in 50% ACN in H_2O and lyophilized.

The lyophilized peptide was dissolved in 50% ACN in water and purified using preparative high-performance liquid chromatography (HPLC) on a Dionex Ultimate 3000 system (Thermo Scientific). Water plus 0.05% TFA and ACN + 0.05% TFA served as buffers A and B, respectively. Gradients from 0% to 65% B over 10 to 45 minutes were applied with a flow rate of 15 mL/min. The peptide mass was confirmed by an LCQ Fleet Ion Trap (Thermo Scientific), and purity was assessed by analytical HPLC (0% to 60% B over 30 minutes, at 1.2 mL/min), using the same solvents as for the preparative HPLC.

On resin N-MeNbz formation: The peptide-N-MeDbz-resin was treated with 4-nitrophenyl chloroformate (5 equiv) in DCM while shaken for 1 hour at room temperature three times. The resin was drained and treated with a 0.5 M DIEA solution in DMF for 30 minutes to complete Nbz formation three times. Finally, the resin was washed with DCM and dried under vacuum.

4. RNF4 sequences

MSTRKRRGGAINSRQAQKRTREATSTPEISLEAEPIELVETAGDEIVDLTCESLEPVVV
DLTHNDSVVIVDERRRPRRNARRLPQDHADSCVVSSDDEELSRDRDVYVTTHTPRN
ARDEGATGLRPSGTVSCPICMDGYSEIVQNGRLIVSTECGHVFCSQCLRDSLKNANT
CPTCRKKINHKRYHPIYI

The Met residues at positions 1 and 135 were replaced with the isologous Norleucine (Nle) residue to prevent oxidation.

5. Synthesis of Fragment-(1a) Cys-RNF4(160–190):

CSQCLRDSLKNANTCPTCRKKINHKRYHPIYI$_{190}$

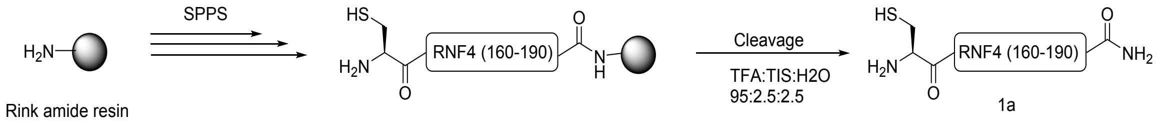

Synthesis was performed on a Rink amide resin (0.27 mmol/g, 0.2 mmol scale). All amino acids were coupled using standard Fmoc-SPPS on the synthesizer to complete the peptide synthesis. The crude peptide 1 was purified by preparative HPLC using a C4 column with a gradient of 0-60% B over 60 minutes. The isolated yield of fragment **(1a)** was approximately 13%.

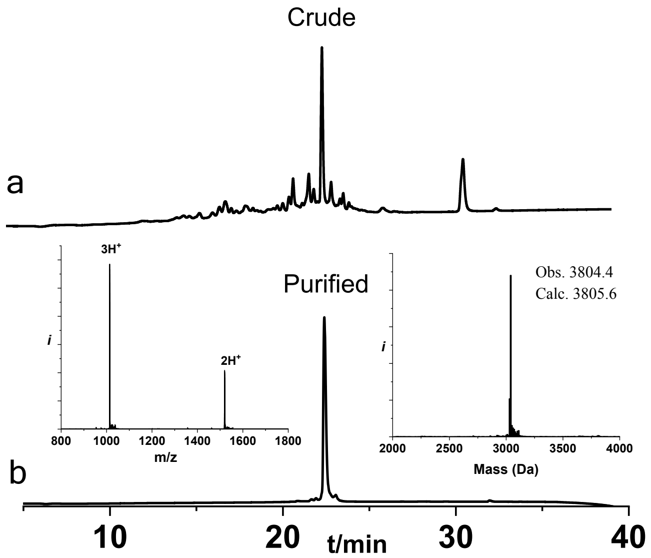

Figure S1. Analytical HPLC of **Cys-RNF4(160-190) (1a):** a) crude; b) purified product **(6)** with an observed mass of 3038.6 Da and a calculated mass of 3039.8 Da.

6. Synthesis of Fragment-(2a) Cys (Acm)-RNF4(133-158)-NHNH$_2$:

CPICNleDG(Dmb)YS EIVQNGRLIVSTECGHVF$_{158}$

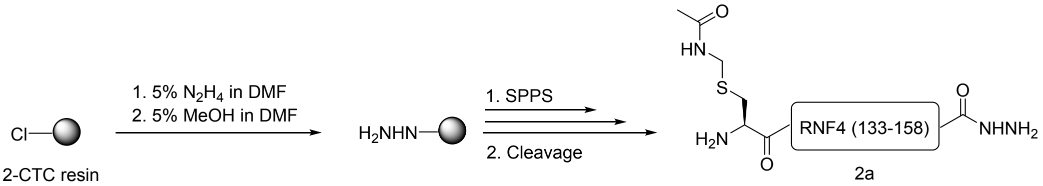

The hydrazide resin was prepared using CTC resin. A 5% hydrazine solution in DMF was added to the resin and incubated for two 45-minute periods. After washing with DMF, DCM, and DMF (three times each), a capping step with 5% MeOH in DMF was performed for 10 minutes. The resin was washed again with DMF, DCM, and DMF (three times each). The first amino acid, Fmoc-Phe-OH (1.0 equivalent), was coupled to the resin using HATU (0.9 equivalents) and DIPEA (2.0 equivalents) in DMF, and the mixture was incubated for 1 hour. The resin was then dried overnight, resulting in a resin loading of 0.32 mmol/gram. The RNF4 fragment 2 sequence was synthesized via standard SPPS, with the last amino acid, Fmoc-Cys (Acm)-OH, coupled. The peptide was cleaved and purified following the standard procedure. The crude peptide 2 was purified using preparative HPLC on a C4 column with a 0-60% B gradient over 60 minutes, The isolated yield of fragment **(2a)** with an isolated yield of 16%.

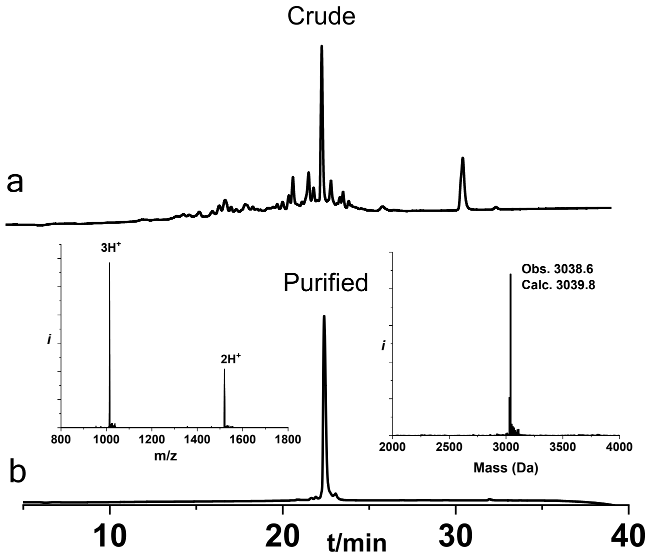

Figure S2. Analytical HPLC of **Cys (Acm)-RNF4(133-158)-NHNH$_2$, (2a)** a) crude; b) purified product with observed mass 3038.6 Da, calculated mass 3039.8 Da.

7. Synthesis of Fragment-(2b) Cys-RNF4(133-158)-NHNH$_2$:

CPICNleDG(Dmb)YS EIVQNGRLIV<u>ST</u>ECGHVF$_{158}$

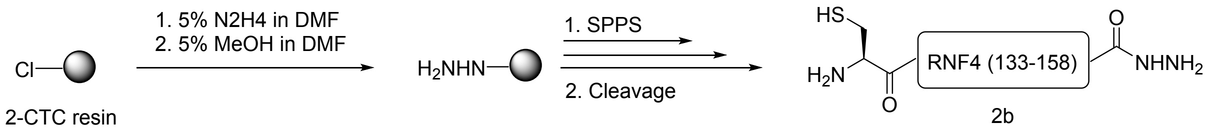

Fragment 2b was synthesized using a 0.2 mmol scale of 2-Cl-(Trt)-Cl resin. For hydrazination, a 5% NH2NH2 solution in DMF was added to the CTC resin and shaken for two cycles, each lasting 1 hour. The resin was then capped with 5% MeOH in DMF to block any remaining reactive chloride groups, followed by washing with DMF and DCM. The first amino acid, serine (Ser), was coupled to the resin. After coupling, the resin was kept for drying overnight. Subsequently, 16.5 mg of the resin was dissolved in a 2% DBU solution, and the absorbance was measured using a Thermo Scientific NanoDrop spectrophotometer. The remaining amino acids were prepared via stepwise Fmoc-SPPS on a 0.2 mmol scale. Synthesis was monitored with HPLC using a C4 column and a gradient from 0 to 60% B over 30 minutes at a flow rate of 1.2 mL. For preparative HPLC, a gradient from 0 to 65% B over 60 minutes at 15 mL/min was used. The isolated yield of fragment **(2b)** was 14%.

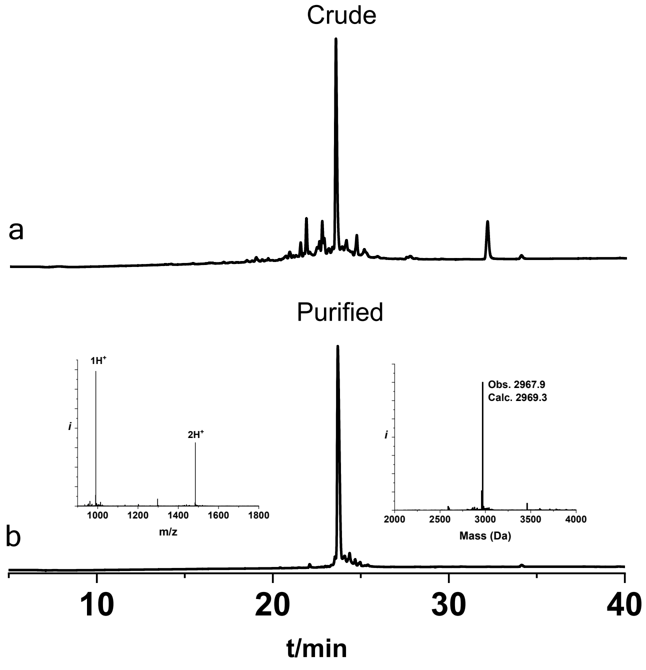

Figure S3. Analytical HPLC of Cys-RNF4(133-158)-NHNH$_2$ (2b): a) crude mixture; b) purified product with an observed mass of 2967.9 Da and a calculated mass of 2969.3 Da.

8. Synthesis of Fragment-(3a) Cys (Acm)-RNF4(92-131)-MMP:

CVVSSDDEELSRDRDVYVTTHTPRNARDEGATGLRPSGTVS$_{131}$

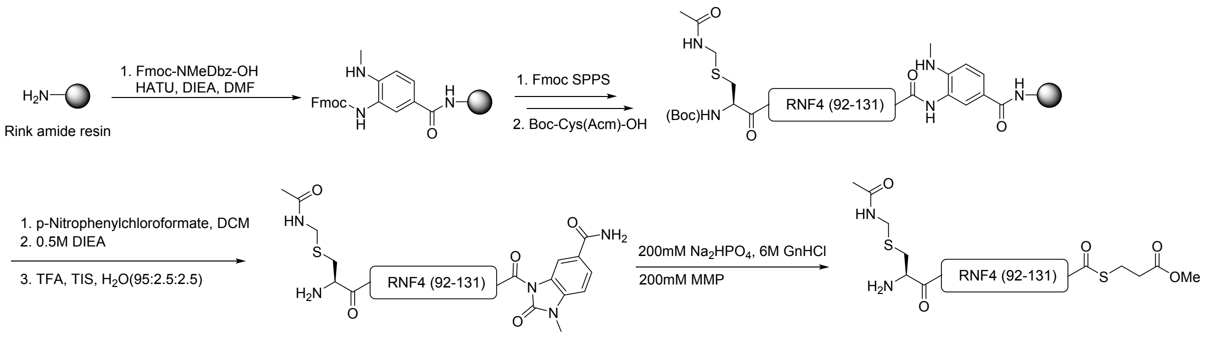

Fragment **3a** RNF4 (92–131)-Nbz was synthesized on pre-swollen Rink amide resin (0.2 mmol) preloaded with Fmoc-NMe-Dbz-OH, following the general procedure. Fmoc-SPPS was performed on the resin, with Boc-Cys (Acm)-OH as the final amino acid. Then, Fragment 3 RNF4 (92–131)-NMe-Dbz-OH was cyclized to the corresponding Nbz derivative according to the general method and subsequently cleaved to produce the crude peptide-Nbz. The peptide-Nbz was converted into the MMP thioester by dissolving it in PB buffer (6 M Gn·HCl, pH ~7), adding 200 mM MMP, and incubating for 1 hour. The crude peptide thioester was purified by preparative HPLC using a C4 column with a 0–65% gradient of buffer B over 40 minutes. The isolated yield of fragment **(3a)** was approximately 12% yield.

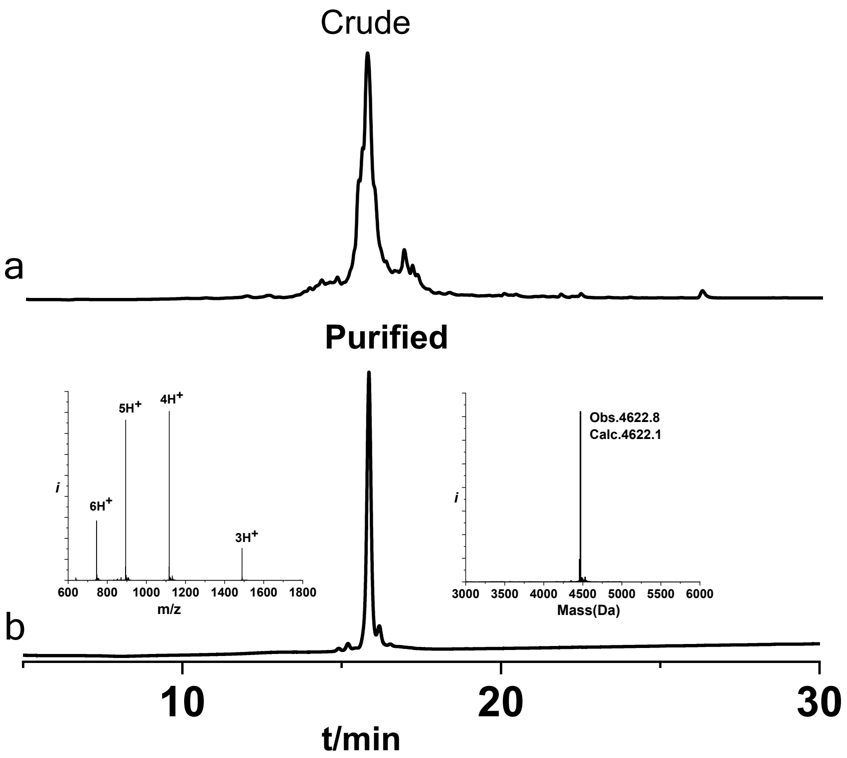

Figure S4. Analytical HPLC of the **Cys (Acm)-RNF4(92-131)-MMP**, **(3a)**: a) crude product, b) purified product with an observed mass of 4622.8 Da and a calculated mass of 4622.1 Da.

9. Synthesis of Fragment-(3b) Cys-RNF4(92-131)- $NHNH_2$:

CVVSSDDEELSRDRDVYVTTHTPRNARDEGATGLRPSGTVS$_{131}$

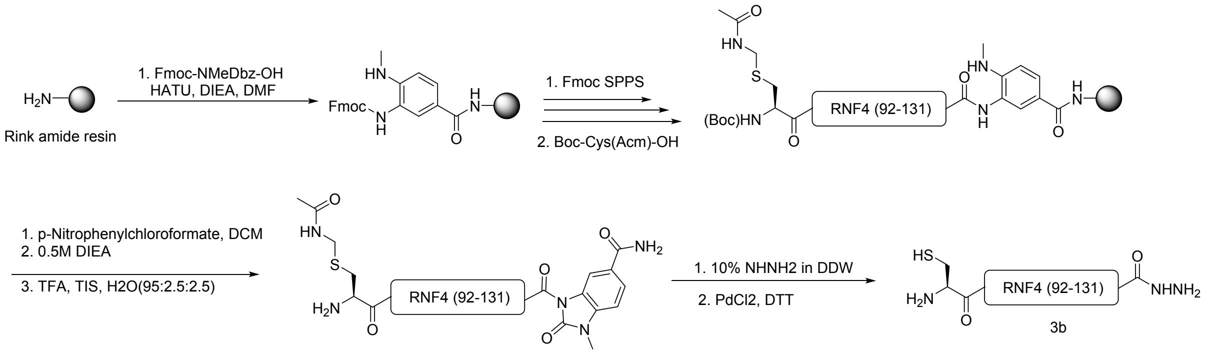

Fragment 3b Cys-RNF4(92–131)-$NHNH_2$ was synthesized on pre-swollen Rink amide resin (0.2 mmol) preloaded with Fmoc-NMe-Dbz-OH, as described in the general procedure. Fmoc-SPPS was carried out on the resin, with Boc-Cys (Acm)-OH as the final amino acid. Fragment 3 Cys (Acm)-RNF4 (92–131)-NMe-Dbz-Resin was then cyclized to the corresponding Nbz derivative according to the general procedure and subsequently cleaved to produce the crude peptide-Nbz. The Nbz moiety was converted to the hydrazine by adding 10% NH_2NH_2 in DDW, and the excess hydrazine was quenched with acetic acid. This was followed by Acm deprotection using $PdCl_2$ and DTT. The crude peptide was purified via preparative HPLC using a C4 column with a 0–65% gradient of buffer B over 40 minutes, yielding the desired hydrazine peptide. The isolated yield of fragment **(3b)** was approximately 8% yield.

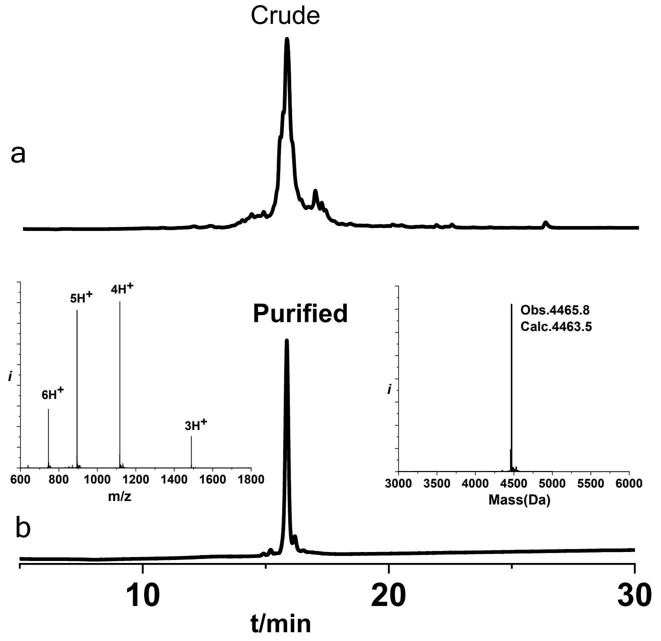

Figure S5. Analytical HPLC of **Cys-RNF4(92-131)-NHNH$_2$ (3b):** a) crude product, b) purified product with an observed mass of 4465.8 Da and a calculated mass of 4463.5 Da.

10. Synthesis of Fragment-(4a) Cys-RNF4(52-90)-$NHNH_2$:

CESLEPVVVDLTHNDSVVIVDERRRPRRNARRLPQDHADS$_{90}$

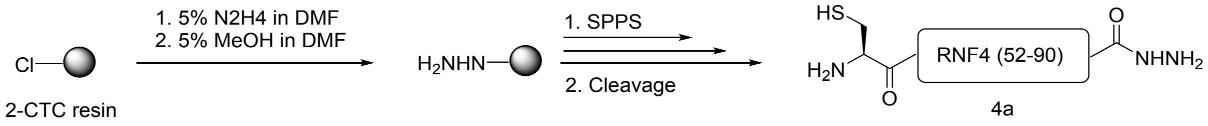

The hydrazide resin was prepared using CTC resin. A 5% hydrazine solution in DMF was added to the resin and incubated for two 45-minute periods. After washing with DMF (3 times), DCM (3 times), and DMF again (3 times), a capping step with 5% MeOH in DMF was performed for 10 minutes. The resin was washed once more with DMF (3 times), DCM (3 times), and DMF (3 times). The first amino acid, Fmoc-Ser-OH (1.0 equiv), was coupled to the resin using HATU (0.9 equiv) and DIPEA (2.0 equiv) in DMF, with the reaction incubated for 1 hour. The resin was dried overnight, resulting in a resin loading of 0.36 mmol/gram. The RNF4 fragment 4a sequence was synthesized via standard SPPS, with the last amino acid, Fmoc-Cys-OH, coupled. The peptide was cleaved and purified in a manner similar to the standard process. The crude peptide 4a Cys-RNF4(52-90)-$NHNH_2$ was purified using preparative HPLC on a C4 column with a gradient from 0 to 60% solvent B over 60 minutes. The isolated yield of fragment **(4a)** was approximately 10%.

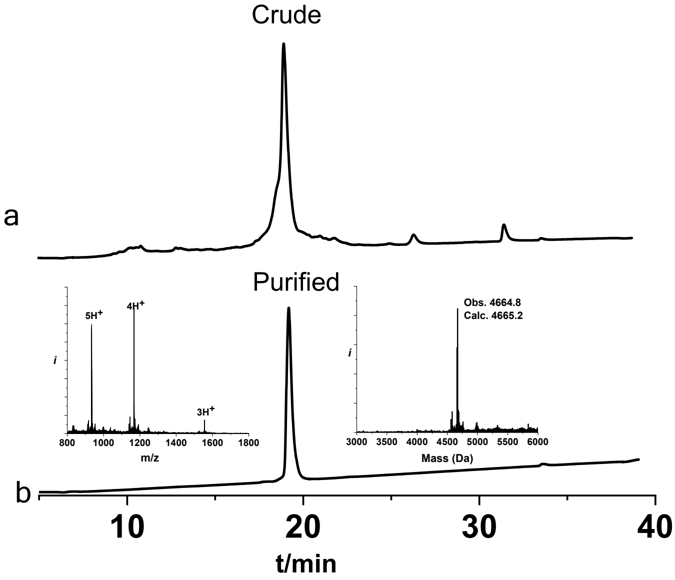

Figure S6. Analytical HPLC of the **Cys-RNF4(52-90)-$NHNH_2$ (4**a) crude sample b) purified product with an observed mass of 4465.8 Da and a calculated mass of 4463.5 Da.

11. Synthesis of Fragment-(5a) Nle-RNF4(1-50)-MMP:

NleSTRKRRGGAINSRQAQKRTREATSTPEISLEAEPIELVETAG(Dmb)DEIVDLT$_{50}$

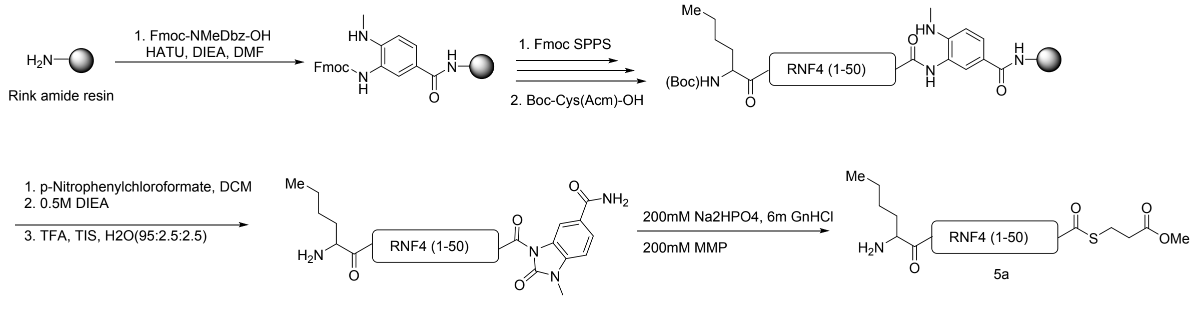

Fragment **5a** Cys-RNF4(1–50)-Nbz was synthesized on pre-swollen Rink amide resin (0.2 mmol) preloaded with Fmoc-NMe-Dbz-OH, as described in the general procedure. Fmoc-SPPS was performed on the resin, with Boc-Nle-OH as the final amino acid. Fragment 5 RNF4 (1–50)-NMe-Dbz-OH was then cyclized to the corresponding Nbz derivative following the general procedure and subsequently cleaved to yield the crude peptide-Nbz. The peptide-Nbz was converted to the MMP thioester by dissolving it in PB buffer (6 M Gn·HCl, pH ~7), adding 200 mM MMP, and incubating for 1 hour. The crude peptide thioester was purified by preparative HPLC using a C4 column with a 0–65% gradient of buffer B over 40 minutes, The isolated yield of fragment **(5a)** was approximately 7%.

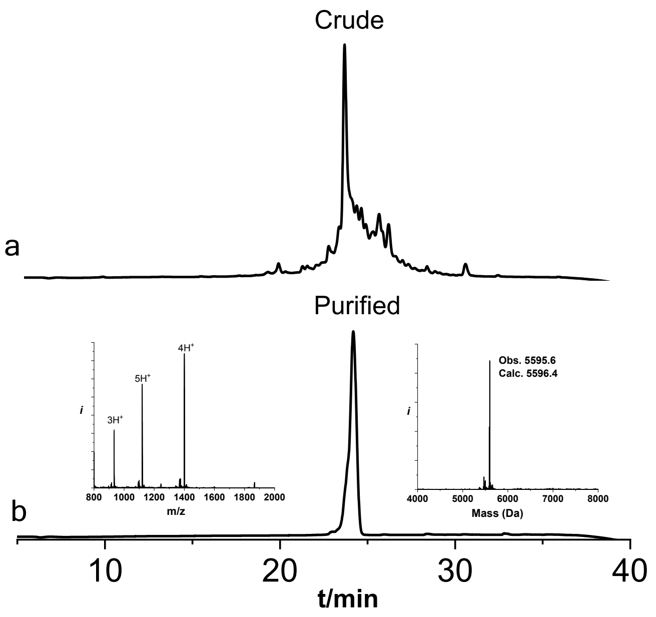

Figure S7. Analytical HPLC and mass data of fragment **Nle-RNF4(1–50)-MMP (5a)**: (a) crude fragment 4, and (b) purified fragment 4 with an observed mass of 5595.6 Da (calculated 5596.4 Da).

12. General procedure for NCL:

The total chemical synthesis of RNF4 was accomplished using five fragments obtained by SPPS, which were subsequently assembled by NCL (native chemical ligation). Each ligation reaction was performed by dissolving 1.0 eq. of the cysteine-containing peptide and 1.3 eq. of the thioester peptide in ligation buffer (6 M Gn·HCl, 200 mM NaPi, 15 eq. TCEP, 25 eq. MPAA, pH 7) to a final concentration of 2 mM for the cysteine peptide. The reactions were carried out at 37 °C and monitored by analytical HPLC.

13. NCL of Fragment 1a and Fragment 2a

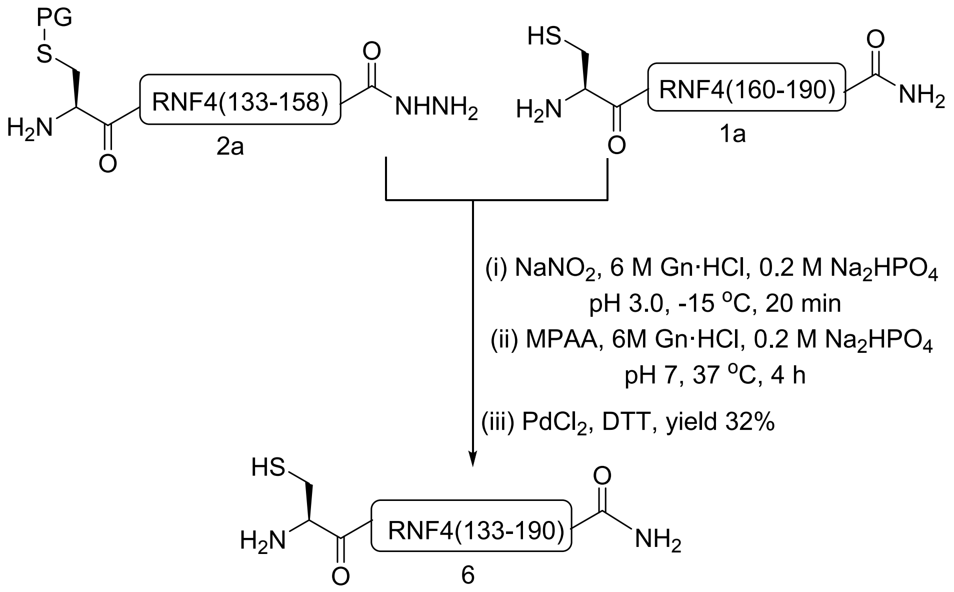

Fragment Cys (Acm)-RNF4(133–158)-NHNH$_2$ (2a), (15 mg, 1.3 equiv), was dissolved in 6 M Gn·HCl and 0.2 M Na_2HPO_4 buffer at pH 3. Then cooled to -15 °C in an ice/salt bath. 40 µL of $NaNO_2$ (15 equiv) was dissolved in water and added to the mixture, which was stirred for 20 min at -15 °C. Afterwards, 57 µL of MPAA (25 equiv) in 6 M Gn·HCl and 0.2 M Na_2HPO_4 buffer at pH 7 were added and gently mixed. Then, **fragment (1a) Cys-RNF4(160-190),** (14.4 mg, 1.0 equiv) was added and gently mixed. The pH of the mixture was adjusted to pH 6.8 using 5 *N* NaOH. After 45 min, 97 µL of TCEP (15 equiv) was added. The reaction progress was monitored by analytical HPLC and MS. After completion, N-terminal Acm was deprotected with 100 µL of $PdCl_2$ (30 equiv) in 6 M Gn·HCl and 200 mM Na_2HPO_4 buffer at pH 7. The mixture was incubated at 37 °C for 1 hour, then quenched with DTT (150 equiv). The mixture was centrifuged, washed with 50% acetonitrile and water, filtered, and purified using RP-HPLC. Affording 8.16 mg of the product **(6) Cys- RNF4(133-190)** at 32% yield.

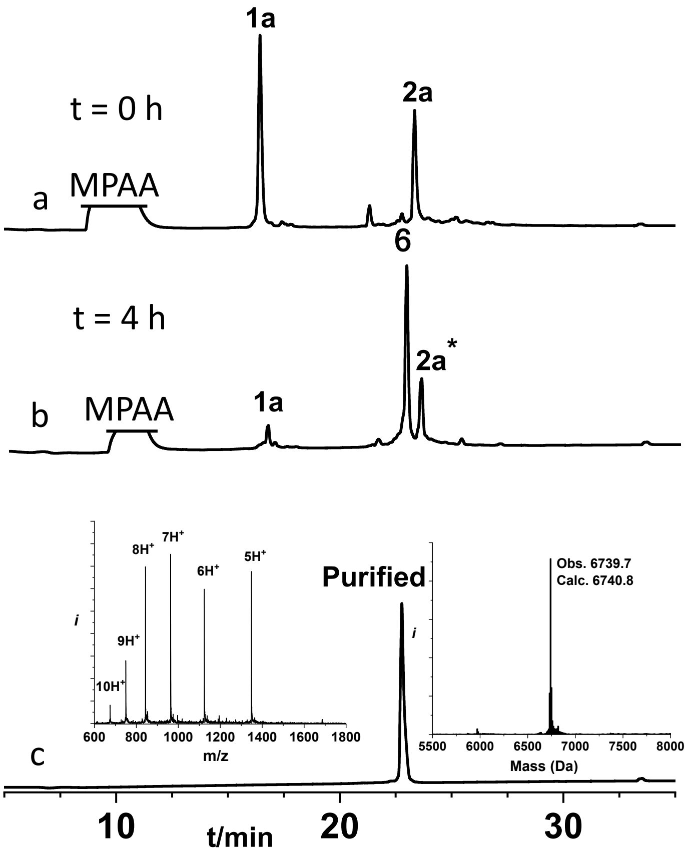

Figure S8. Analytical HPLC of the ligation reaction: a) at 0 minutes, b) after 4 hours, and c) purified product (**6**), **Cys-RNF4(133-190),** with an observed mass of 6739.7 Da and a calculated mass of 6740.8 Da.

14. NCL of peptide 6 and fragment 3a

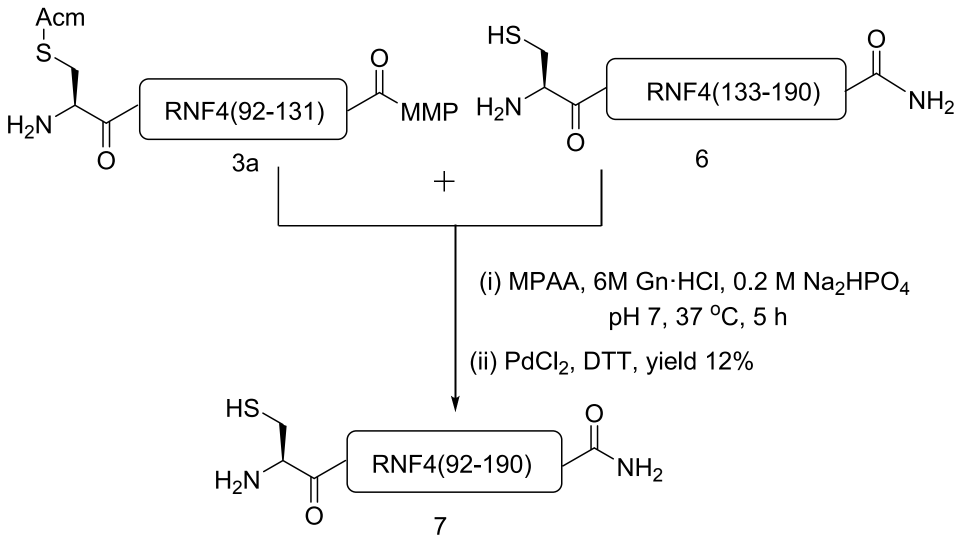

Peptide (6) Cys-RNF4(133-190) (8 mg, 1.0 equiv) was dissolved in 593 μL of 6 M Gn·HCl, 0.2 M Na_2HPO_4 buffer at pH 7 containing a mixture of TCEP (15 equiv) and MPAA (25 equiv). Then, **fragment (3a) Cys (Acm)-RNF4(92-131)-MMP** was added to the reaction mixture and incubated for 5 hours until the ligation was complete. The reaction was monitored using analytical HPLC and LC-MS. After the reaction was complete, N-terminal Acm was deprotected by the addition of 100 μL $PdCl_2$ (30 equiv) in 6 M Gn·HCl, 200 mM Na_2HPO_4 buffer at pH 7. The mixture was incubated at 37°C for 1 hour and then quenched with DTT (150 equiv). The mixture was centrifuged, washed with 50% acetonitrile and water, filtered, and purified using RP-HPLC, affording 1.85 mg of the product **(7) Cys-RNF4(92-190)** (12% yield).

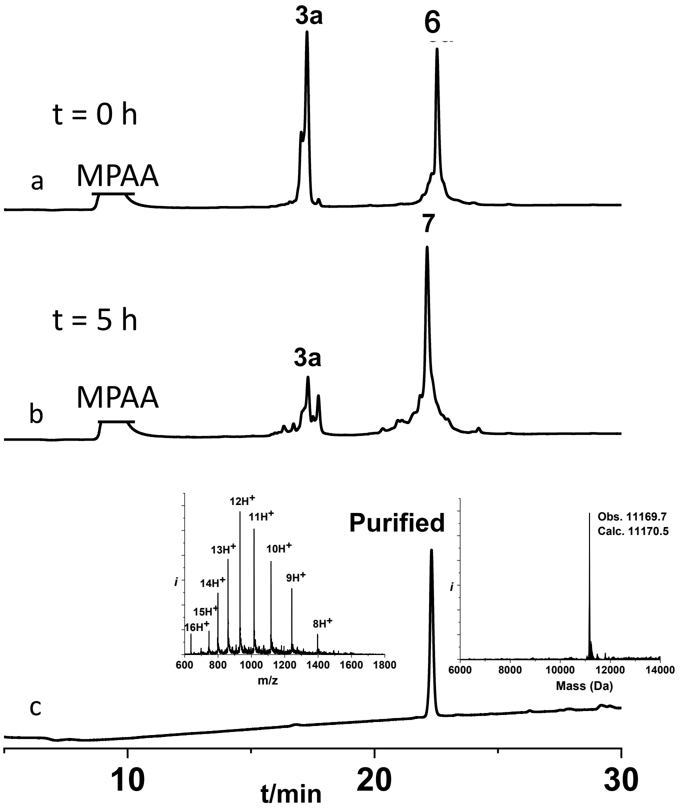

Figure S9. Analytical HPLC of the ligation reaction: a) at 0 min, b) after 4 hours, and c) purified product **(7) Cys-RNF4(92-190)** with an observed mass of 11169.7 Da and a calculated mass of 11170.5 Da.

15. NCL of Fragment 4a and Fragment 5a

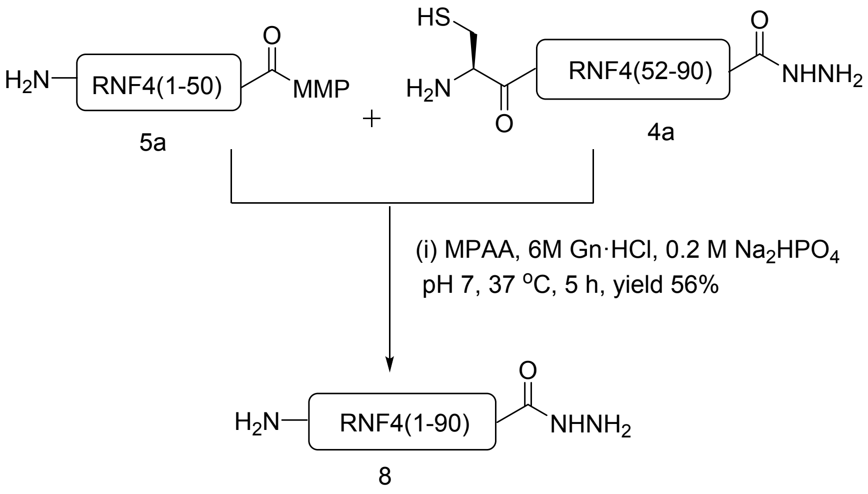

Fragment (4a) Cys-RNF4(52-90)-NHNH$_2$ (10 mg, 1.0 equiv) was dissolved in 1 mL of 6 M Gn·HCl and 0.2 M Na_2HPO_4 buffer at pH 7 containing a mixture of TCEP (15 equiv) and MPAA (25 equiv). Then, **fragment (5a) RNF4(1-50)-MMP** was added to the reaction mixture and incubated for 5 hours until ligation was complete. The reaction was monitored using analytical HPLC and LC-MS. After the reaction was complete, purification was carried out using RP-HPLC, affording 12.2 mg of the product **(8) RNF4(1-90)-NHNH$_2$** (56% yield).

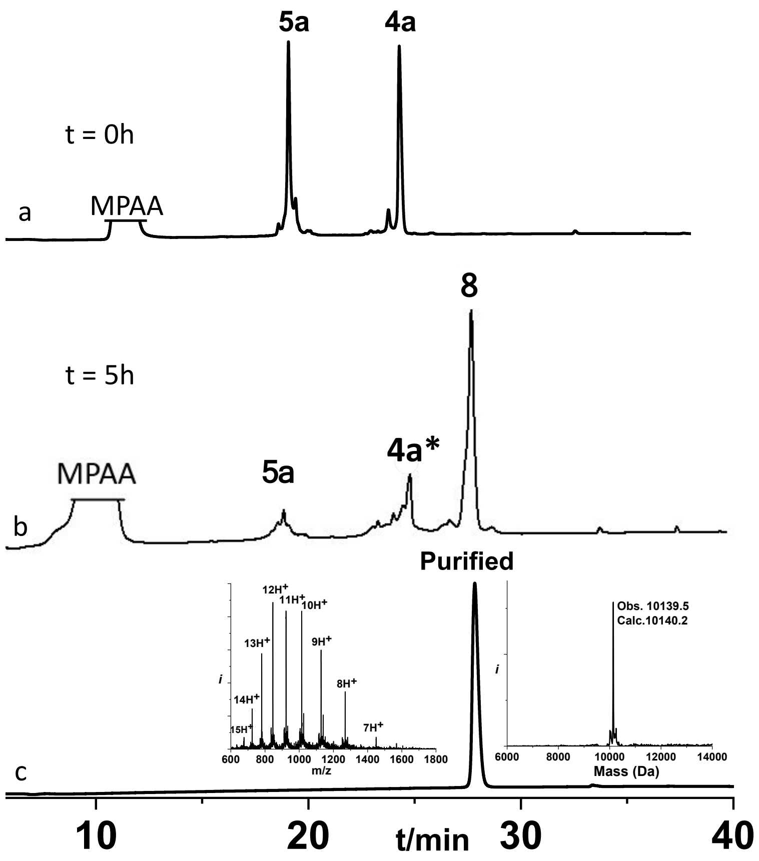

Figure S10. Analytical HPLC reaction of the ligation: a) at 0 minutes, b) after 5 hours, and c) the purified product **(8) RNF4(1-90)-NHNH$_2$** with an observed mass of 10139.5 Da and a calculated mass of 10140.2 Da.

16. NLC of peptide 7 with peptide 8

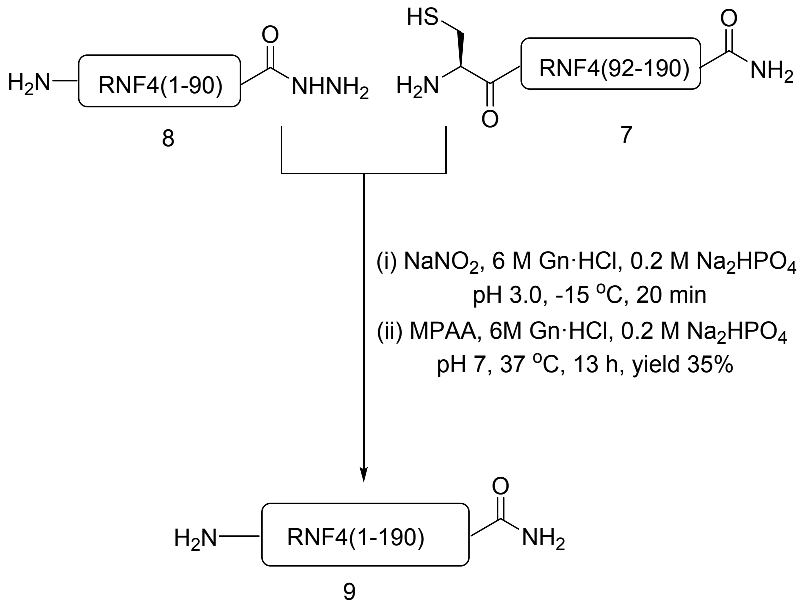

Fragment **(8) RNF4(1-90)-NHNH$_2$** (1.65 mg, 1.3 equiv) was dissolved in 6 M Gn·HCl and 0.2M Na_2HPO_4 buffer at pH 3. Then, it was cooled to -15 °C with an ice/salt bath. 9 µL of $NaNO_2$ (15 equiv) was added to the reaction mixture and reacted for 20 min at -15 °C. After 20 minutes, 15 µL of MPAA (50 equiv) in 6 M Gn·HCl and 0.2 M Na_2HPO_4 buffer at pH 7 were added and gently mixed. Then, peptide **(7) Cys-RNF4(92-190),** (1.85 mg, 1.0 equiv) was added and gently mixed. The pH of the mixture was adjusted to pH 6.8 using 5 *N* NaOH. After 45 minutes 33 µL of TCEP (15 equiv) was added. Analytical HPLC and MS monitored the progress of the reaction. After the completion of the reaction, purification was carried out using RP-HPLC, affording 1.2 mg of the final product **(9) RNF4(1-190)** 35% yield.

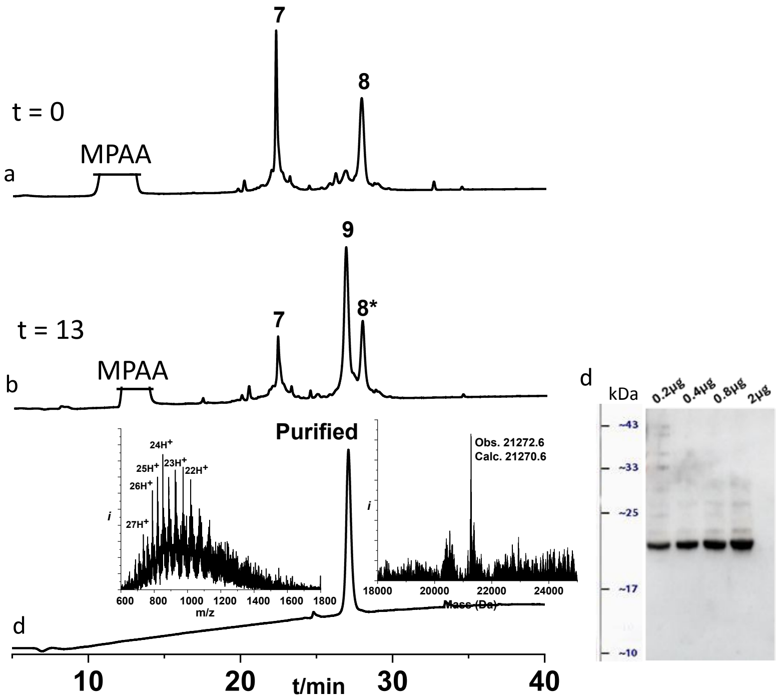

Figure S11. Analytical HPLC of the ligation reaction: a) at 0 minutes, b) after 6 hours, and c) HPLC and mass spectrometric analysis, observed mass 21272.6 Da (calculated 21,270.6 Da). d) Western blot analysis using anti-RNF4.

17. One-pot ligation of fragments 5a-3b

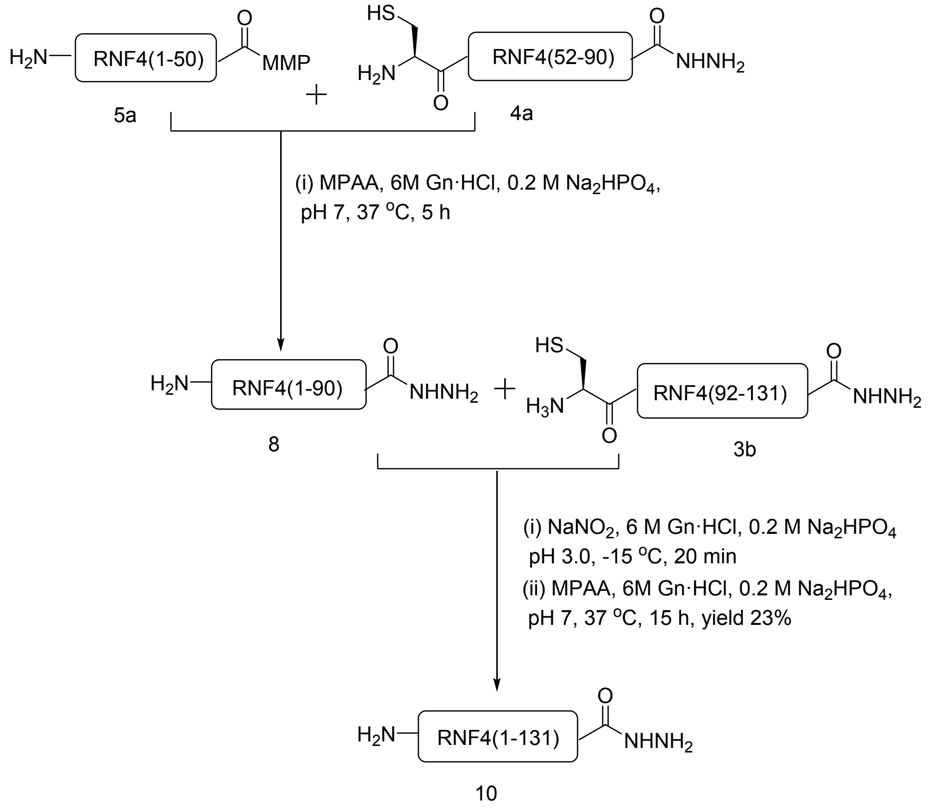

Fragment (4a) Cys-RNF4(52-90)-NHNH$_2$ (5 mg,1 equiv) was dissolved in a mixture of TCEP (30 equiv) and MPAA (25 equiv) dissolved in 6M Gn·HCl, 0.2 M Na_2HPO_4 buffer at pH 7. Then, **fragment (5a) RNF4(1-50)-MMP** (1 equiv) was dissolved in the reaction mixture and incubated for 5h until the ligation was complete. The reaction was monitored using analytical HPLC and LC-MS. After 5h ligation, the reaction mixture was desalted into a 3KDa molecular weight cutoff spin filter (Vivaspin®500 -0.5mL). The reaction mixture was centrifuged at 1000 rpm for 30 min. Subsequently next ligation between intermediate peptide **8** and fragment **3b**. The pH of product **8** was then adjusted to pH 3 using 5 *N* HCl. Then cooled to -15 °C by using an ice/salt bath. 32µL of $NaNO_2$ (20 equiv) was dissolved in water and added to the reaction mixture, and reacted for 20 min at -15 °C. After 20 min, 100 µL of MPAA (50 equiv) in 6M Gn·HCl, 0.2 M Na_2HPO_4 buffer at pH 7, was added. The pH of the mixture was adjusted to pH 6.8 using 5 *N* NaOH. **Fragment (3b) Cys-RNF4(92-131)-NHNH$_2$** (1 equiv) was added to the mixture and gently mixed. After 45 min,115 µL of TCEP (30 equiv) was added to the reaction mixture. Analytical HPLC and MS. monitored the progress of the reaction. After the completion of the reaction, purification was carried out using RP-HPLC, affording 3mg of the final product **(10) RNF4(1-131)-NHNH$_2$** (23% yield).

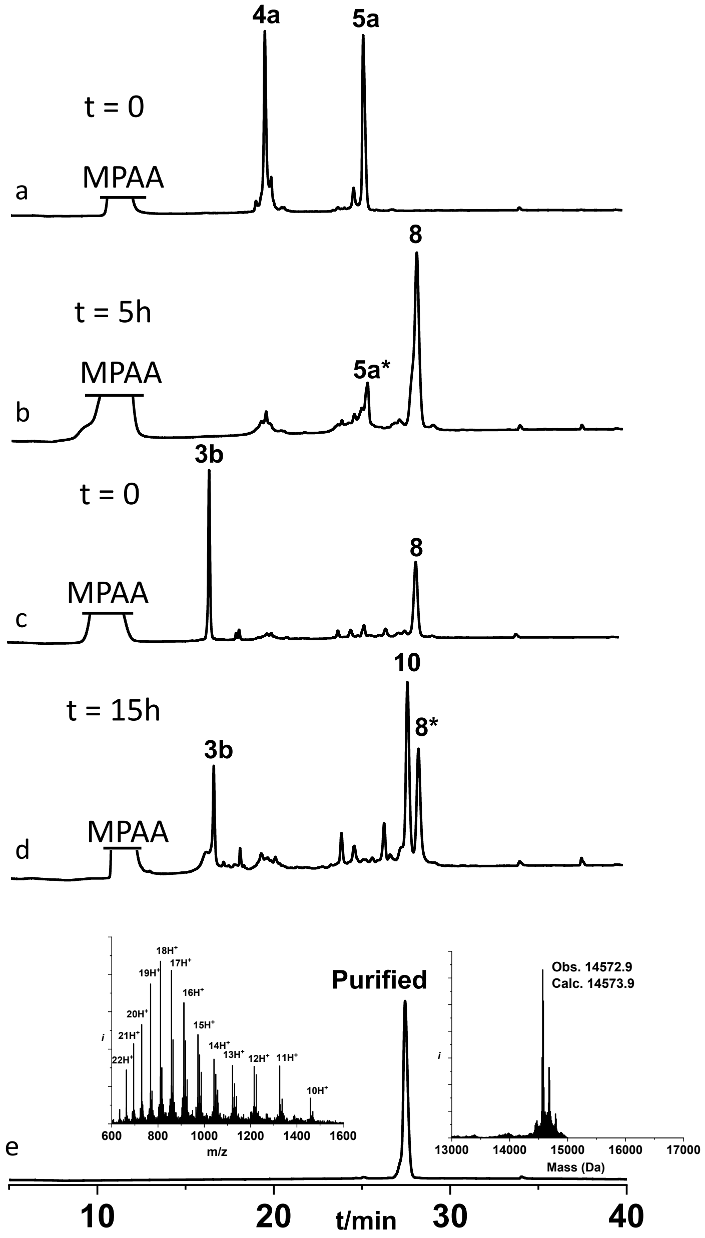

Figure S12. Analytical HPLC of the one-pot ligation reaction: (a) first ligation at t = 0 min; (b) crude first ligation reaction at t = 5h, product **(8) RNF4(1-90)-NHNH$_2$**, hydrolysis of RNF4 (1-50) thioester (5a*); (c) second ligation at t = 0 min; (d) crude second ligation reaction at t = 15h, product **(10) RNF4(1-131)-NHNH$_2$**, hydrolysis of RNF4 (1-90) thioester (8a*); (e) purified product **(10) RNF4(1-131)-NHNH$_2$** with observed mass 14572.9 Da, calculated mass 14573.9 Da.

18. NCL of Peptide 10 and fragment 2b:

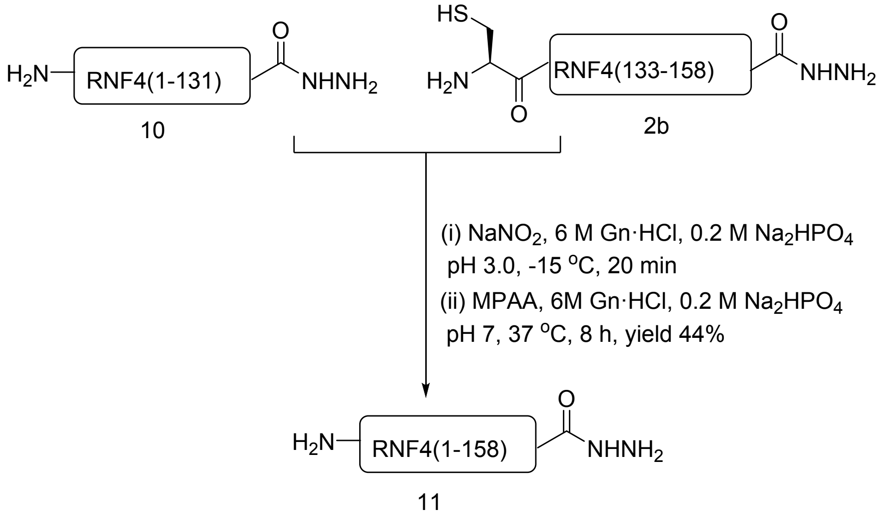

RNF4(1-131)-NHNH$_2$ (10) (3mg, 1 equiv) was dissolved in 6M Gn·HCl, 0.2 M Na_2HPO_4 buffer at pH 3, cooled to -15 °C by using an ice/salt bath. 6.26µL of $NaNO_2$ (15 equiv) was added to the reaction mixture, and reacted for 20 min at -15 °C. After 20 min, 50 µL of MPAA (50 equiv) in 6M Gn·HCl, 0.2 M Na_2HPO_4 buffer at pH 7, was added to the mixture and gently mixed. The pH of the mixture was adjusted to pH 6.8 using 5 *N* NaOH. Then **Cys-RNF4(133-158)-NHNH$_2$ (2b)** (0.3mg, 0.5 equiv) was added to the mixture. After 45 min,32 µL of TCEP (30 equiv) was added to the reaction mixture. Analytical HPLC and MS monitored the progress of the reaction. After the completion of the reaction, purification was carried out using RP-HPLC, affording 1.6 mg of the final product **(11) RNF4(1-158)-NHNH$_2$** (44% yield).

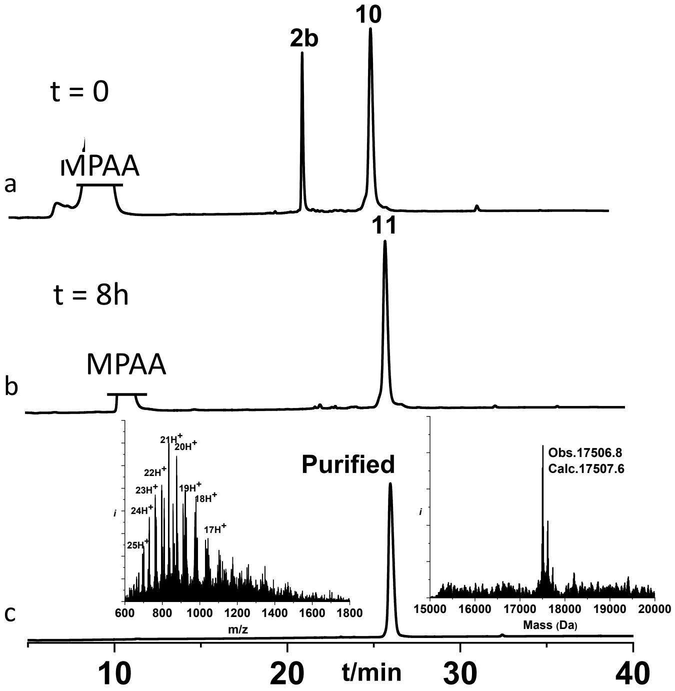

Figure S13. Analytical HPLC of the ligation reaction: a) at 0 minutes and b) after 8 hours, and (c) c) purified product **(11) RNF4(1-158)-NHNH$_2$** with an observed mass of 17506.8 Da and a calculated mass of 17507.6 Da.

19. NCL of peptide 11 and fragment 1a

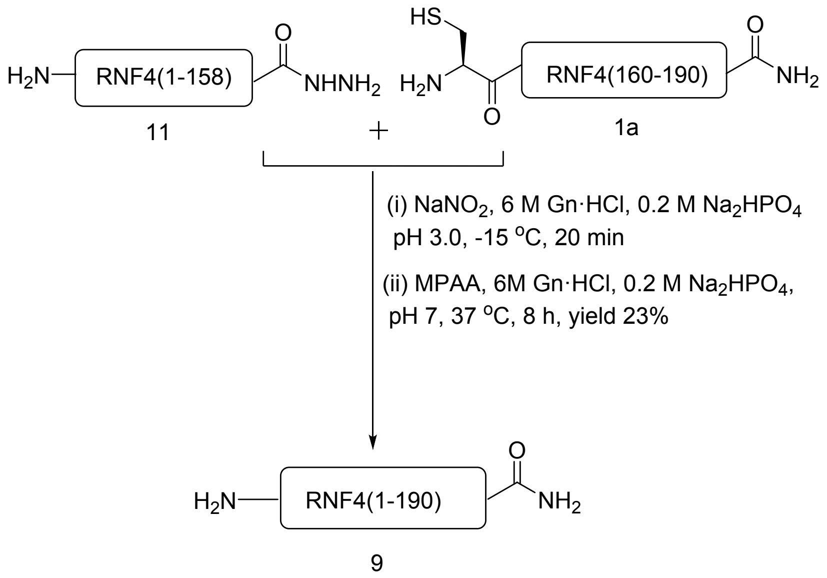

RNF4(1-158)-$NHNH_2$ (11) (1.6 mg, 1equiv) was dissolved in 6M Gn·HCl, 0.2 M Na_2HPO_4 buffer at pH 3. Then cooled to -15 °C by using an ice/salt bath. 41µL of $NaNO_2$ (15 equiv) was dissolved in water and added to the reaction mixture, and reacted for 20 min at -15 °C. After 20 min, 50 µL of MPAA (50 equiv) in 6M Gn·HCl, 0.2 M Na_2HPO_4 buffer at pH 7, was added to the mixture and gently mixed. The pH of the mixture was adjusted to pH 6.8 using 5 *N* NaOH. Then **Cys-RNF4(160-190)-$NHNH_2$ (1a)** (0.5mg, 1 equiv) was added to the mixture and gently mixed. After 45 min,19 µL of TCEP (30 equiv) was added to the reaction mixture. Analytical HPLC and MS monitored the progress of the reaction. After completion of the reaction, purification was carried out using RP-HPLC, affording 0.45 mg of the final product **(9) RNF4(1-190) (9)** (23% yield).

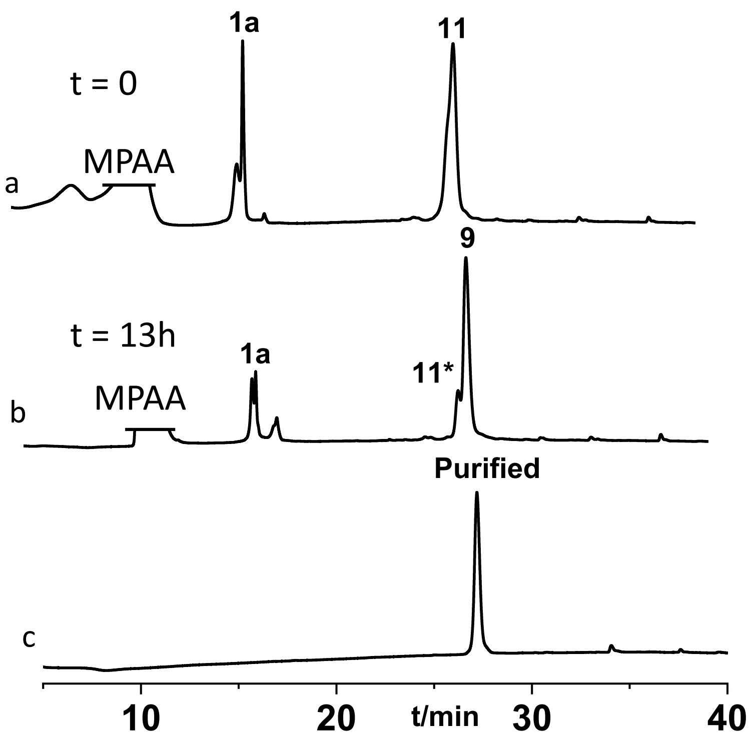

Figure S14. Analytical HPLC of the ligation reaction: a) at time 0 min, and b) after 13 hours, unmodified mass (*). c) Purified final product RNF4(1-190) **(9)** with an observed mass of 21272.6 Da, and a calculated mass of 21270.6 Da.
